# Reappraisal of the thalattosuchian crocodylomorph record from the Middle-Upper Jurassic Rosso Ammonitico Veronese of northeastern Italy: Age calibration, new specimens and taphonomic biases

**DOI:** 10.1371/journal.pone.0293614

**Published:** 2023-10-30

**Authors:** Giovanni Serafini, Davide Foffa, Mark T. Young, Giacomo Friso, Miriam Cobianchi, Luca Giusberti

**Affiliations:** 1 Dipartimento di Scienze Chimiche e Geologiche, Università degli Studi di Modena e Reggio Emilia, Modena, Italy; 2 Department of Geosciences, Virginia Tech, Blacksburg, Virginia, United States of America; 3 School of Geography, Earth and Environmental Sciences, University of Birmingham, Birmingham, United Kingdom; 4 Department of Natural Sciences, National Museum of Scotland, Edinburgh, United Kingdom; 5 School of Biological Sciences, Faculty of Environmental and Life Sciences, University of Southampton, Southampton, United Kingdom; 6 LWL-Museum für Naturkunde, Münster, Germany; 7 Dipartimento di Geoscienze, Università degli Studi di Padova, Padova, Italy; 8 Dipartimento di Scienze della Terra e dell’Ambiente, Università degli Studi di Pavia, Pavia, Italy; Universidad de Magallanes, CHILE

## Abstract

Despite their extremely rare and fragmentary record, aquatic crocodylomorphs from the Middle to Upper Jurassic (Bajocian-Tithonian) Rosso Ammonitico Veronese (RAV) of northeastern Italy have sparked interest since the late 18th century. Among marine reptiles, Thalattosuchia is by far one of the best represented groups from the RAV units, especially in the Middle Jurassic. Although some specimens have been the subject of multiple studies in recent times, most of them still lack precise stratigraphic assignment and taphonomic assessment, while others remain undescribed. Here we provide a comprehensive revision of the thalattosuchian record from the RAV, alongside the most up-to-date age determination, by means of calcareous nannofossils, when available. Three new metriorhynchoid specimens are described for the first time from the Middle Jurassic of Asiago Plateau (Vicenza province). While the taphonomy of the newly described specimens hampers any taxonomic attribution below superfamily/family level, all three were confidently assigned to a precise interval between the upper Bajocian and the upper Bathonian. This revised record has major paleobiogeographical implications: the new specimens confirm an early origin and distribution of Metriorhynchoidea in the Tethys area and suggest a fast colonization of the open-ocean environment since the upper Bajocian.

## Introduction

Thalattosuchia was an aquatic clade of crocodylomorphs known from the Jurassic and Early Cretaceous (e.g., [1–8]). Within Thalattosuchia, two subclades are recognized, Metriorhynchoidea and Teleosauroidea [9, 10]. Teleosauroidea was a clade of predominantly long-snouted forms superficially similar to modern gharials (e.g., [2, 3]). However, teleosauroids were ecologically and anatomically diverse, for example the short-snouted machimosaurin machimosaurids had dentition specialized for a durophagous or chelonivorous diet, while aeolodontin teleosaurids had postcranial adaptations for a more aquatic lifestyle (see [10–13]). It is within Metriorhynchoidea that we see the transition from semi-aquatic species into fully pelagic forms. Although early diverging metriorhynchoids remain poorly understood, a rapid shift to a more aquatic body plan can be observed since the Middle Jurassic. These changes include: the verticalization of the orbits, enlarged salt glands, the flattening of the ulnae and the beginnings of a hypocercal tail (e.g., [3, 14–17]). This macroevolutionary trend reached its peak with Metriorhynchidae. The aquatic specializations of metriorhynchids were extensive, ranging from their compact inner ears [18] to reduced girdles [1, 2, 19–22], hydropedal limbs [1, 19], flexural caudal vertebrae supporting a true hypocercal tail [1, 21, 22] and smooth body integument, lacking both scales and osteoderms [1, 23]. While major evolutionary radiations for thalattosuchians are well documented in epicontinental seas from Central and Northern Europe (e.g., Toarcian diversification of teleosaurids from Germany and France and Oxfordian-Kimmeridgian radiation of metriorhynchids from the UK and France; [4, 10, 24]), the paleobiogeography of the group in the Tethys Ocean is otherwise poorly known [4, 25].

Starting from the late 18th century, the Rosso Ammonitico Veronese (RAV) from Veneto region of northern Italy (Bajocian-Tithonian; [26]) has yielded several marine reptile specimens, mostly in poor state of preservation and of difficult taxonomical interpretation [25–34]. Despite strong preservational biases, tetrapods from the RAV represent a unique window in the paleoecology of the westernmost European side of the Tethys during the Middle-to-Upper Jurassic. This area, in fact, remains largely undersampled in the Bajocian-Oxfordian interval [24], compared to its Central European and British counterparts. Currently 23 marine reptile specimens (counting isolated elements as single individuals) have been discovered from RAV units including ichthyosaurs [29, 33], plesiosaurs [30, 32, 35], metriorhynchoids [31, 36] and a single aeolodontin teleosauroid [28, 34]. Other representatives of Thalattosuchia from Italy include two putative *Pelagosaurus* specimens from the Calcare di Sogno (Lower Jurassic of Cesana Brianza, Lecco province, Lombardy; [37]), isolated teeth from the Rotzo Formation (Lower Jurassic of the Trentino Alto Adige region; [38, 39]) and from the Toarcian-?Bajocian Encrinite of Monte Verzegnis (Friuli-Venezia Giulia region; [40]), a metriorhynchid partial rostrum from the Maiolica (lower Berriasian of Asiago Plateau; [41]) and a single metriorhynchid tooth from the Lower Cretaceous of Sicily [5, 42]. In this context, the thalattosuchians from the RAV, with a total of 9 specimens (8 individuals) that we report here, represent a significant proportion of the Jurassic marine tetrapod fossil record in Italy. This record includes three previously undescribed specimens of metriorhynchoids from three separate localities that were re-discovered during museum collection surveys. Although the subject of recent taxonomical and time-calibrated phylogenetic studies [25, 35, 31], the RAV metriorhynchoid record is still in need of precise biostratigraphic ordering, and of a detailed taphonomic analysis of the main biostratinomic processes that specimens might have experienced. Here, we revise the thalattosuchian record from the RAV of the Southern Alps, with particular focus on the stratigraphic position and preservation of each specimen and the first apomorphy-based [43, 44] description of the new material.

## Geological setting

**Fig 1:**
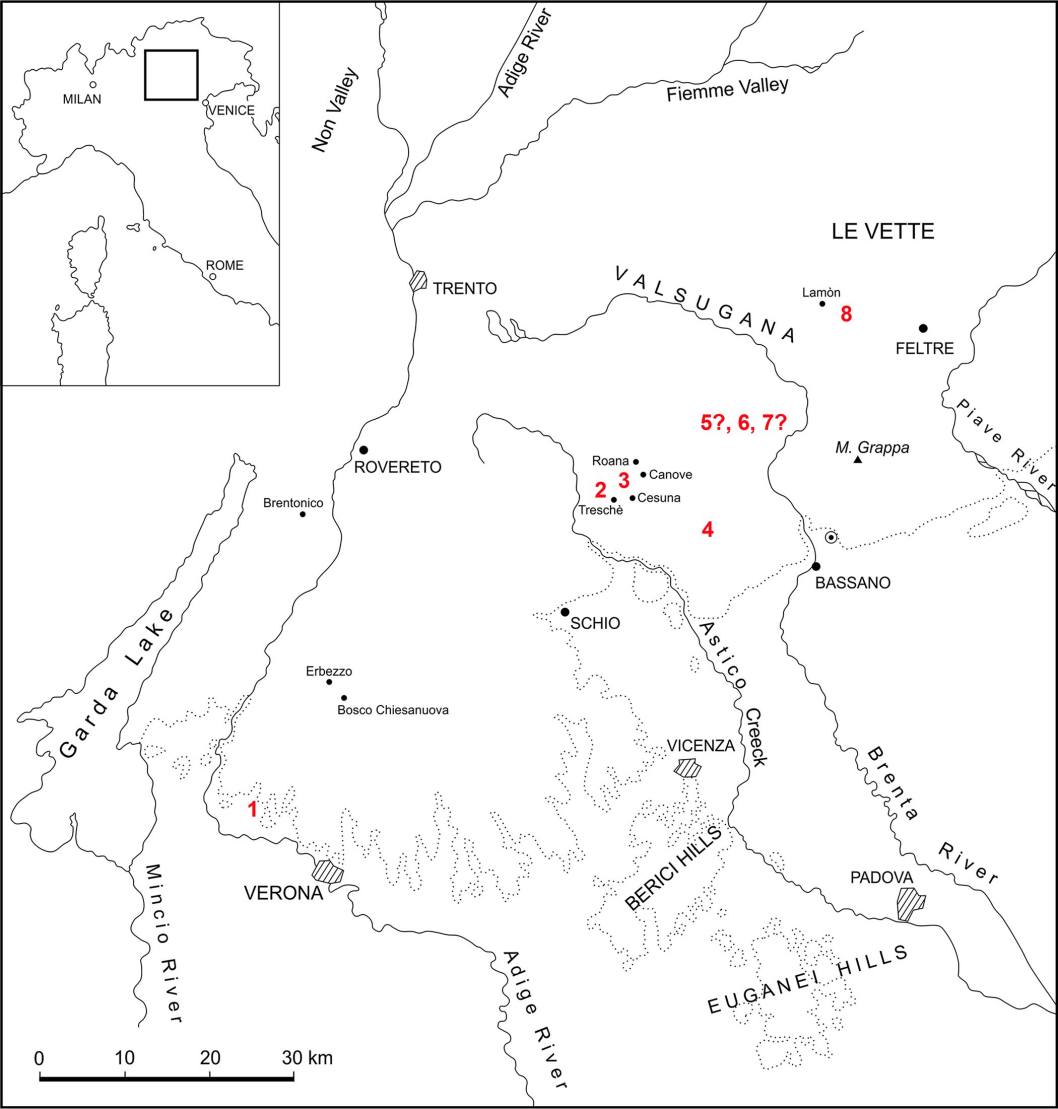
Location map of the study area in northeastern Italy with indication of the localities that yielded thalattosuchian remains from the Middle-Upper Jurassic Rosso Ammonitico Veronese. Numeration is arbitrarily started from the left; it does not match the order of discovery. 1) S. Ambrogio di Valpolicella (Verona province), possible source locality of the *Neptunidraco ammoniticus* holotype. 2) Tresché Conca (Vicenza province), source locality of MGP-PD 26552. 3) Cesuna (Vicenza province), source locality of MGP-PD 6572 and MGP-PD 6761. 4) Cima del Porco (Asiago, Vicenza province), source locality of MGP-PD 32438. 5) Unknown source locality of MM 25.5.1078 in the Asiago area. 6) Sasso d’Asiago (Vicenza province) source locality of FMCR SP3839. 7) Sasso d’Asiago (Vicenza province), possible source locality of MCLSC T2. 8) Ponte Serra (Belluno province), source locality of MGP-PD 27566. Map modified and reprinted from [50] under a CC BY license, with permission from Bollettino della Società Paleontologica Italiana, original copyright year 1981.

**Fig 2:**
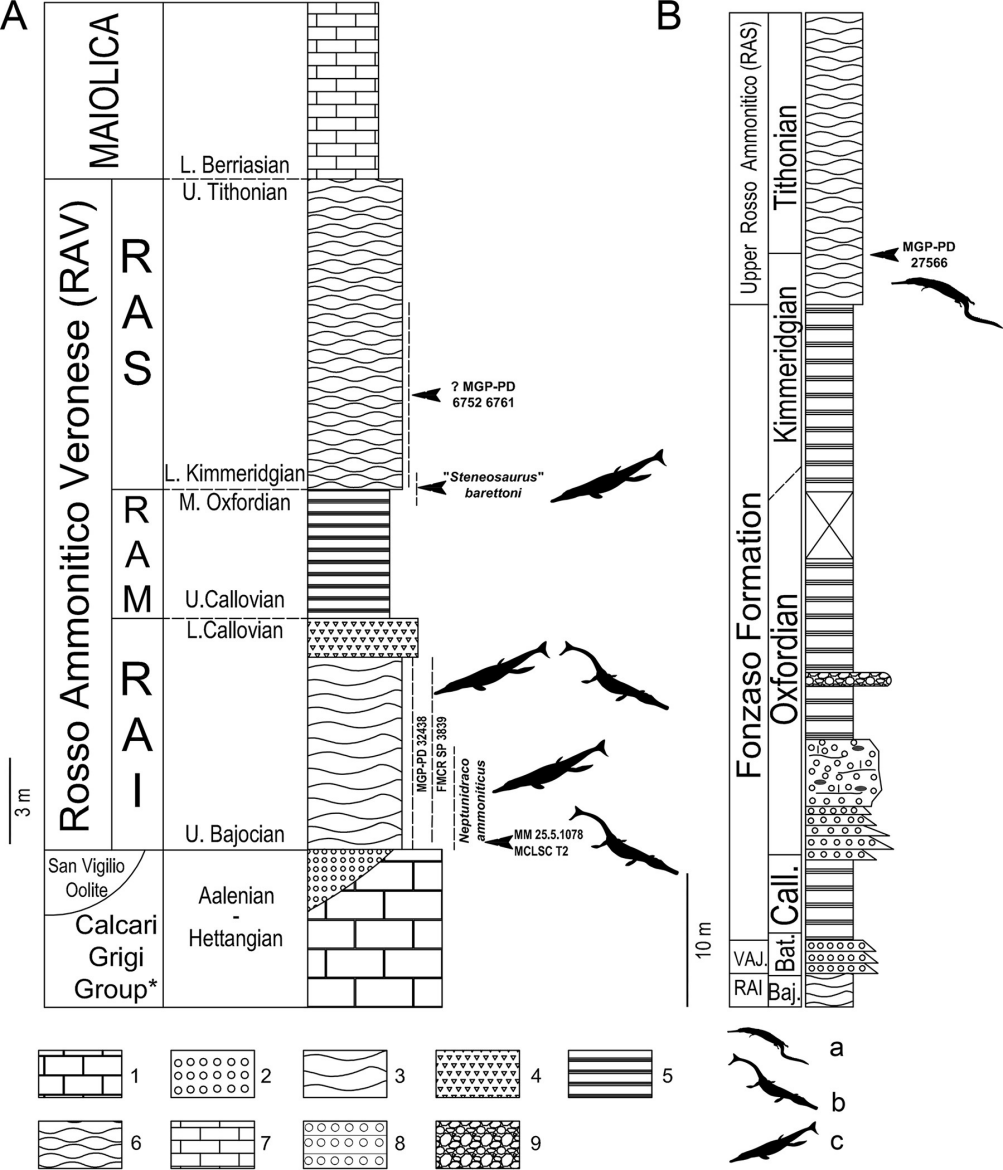
(A) Idealized stratigraphy of the Rosso Ammonitico Veronese (RAV) in the Trento Plateau (Verona area and Asiago Plateau), and (B) the Middle-Upper Jurassic stratigraphic succession of Ponte Serra (Belluno) with inferred stratigraphic position of each crocodylomorph specimen so far recovered in the RAV and herein discussed. Silhouettes: a) Teleosauroidea (CC BY 3.0 Gareth Monger); b) Metriorhynchoidea (CC BY 3.0 Gareth Monger); c) Metriorhynchidae (CC BY 3.0 Mette Aumala). Lithostratigraphic and lithologic legend: 1) Hettangian-Domerian shallow-water limestones of the Calcari Grigi Group (Asiago Plateau); 2) Toarcian-Aalenian oolithic limestones of the San Vigilio Oolite (Lessini Mountains); 3) pseudonodular and 4) bioclastic limestones of the Bajocian p.p.-Callovian p.p. Rosso Ammonitico Inferiore (RAI); 5) well bedded cherty limestones (Callovian p.p.-Oxfordian p.p. Rosso Ammonitico Medio, RAM and Bathonian p.p.-Kimmeridgian p.p. Fonzaso Formation); 6) nodular limestones of the Kimmeridgian p.p.-Tithonian Rosso Ammonitico Superiore (RAS); 7) micritic cherty limestones of the Lower Cretaceous Maiolica; 8) oolitic and peloidal grainstones/packstones; 9) synsedimentary breccia. Compiled based on various sources [34, 47, 51].

Our specimen sample comes from three different areas of the Veneto region in the Southern Alps of Italy (Fig 1): the Asiago Plateau (“Altopiano di Asiago”, Vicenza province), the S. Ambrogio di Valpolicella area (Lessini Mountains, Verona province) and Ponte Serra (western Belluno province). During the Early Jurassic these areas were part of the Trento Platform, a vast Hettangian-Aalenian carbonate platform that was delimited by deeper basins, namely westward by the Lombardian Basin and eastward by the Belluno Trough [45]. In the Middle Jurassic, this structural high transformed in an articulated current-swept plateau (Trento Plateau) with greatly reduced pelagic sedimentation (a few millimeters per kyr), giving rise to the so-called “Rosso Ammonitico Veronese” deposits (RAV; [46]). The drowning of the Trento Platform is marked by a regional unconformity separating the shallow water facies of the Calcari Grigi Group (Asiago area) and more open marine deposits of the San Vigilio Oolite (Verona area) from the overlying limestones of RAV [26]. At the top of Calcari Grigi in the Asiago Plateau, bivalve-and ammonite-bearing coquinas are locally present, mainly as fillings of cavities (e.g., [26]). The overlying Middle-Upper Jurassic Rosso Ammonitico Veronese (RAV), up to 30 m thick, is a very distinctive lithostratigraphic unit in the Mesozoic succession of Trento Plateau and mostly consists of commonly nodular pink to red ammonite-bearing limestones gradually grading to micritic white limestones belonging to the uppermost Jurassic-Lower Cretaceous Maiolica [47]. The RAV is characterized by marked lateral variations of facies and thickness due to irregular topographies on the Trento Plateau, and usually shows a typical tripartition into three members, the Rosso Ammonitico Inferiore (RAI, upper Bajocian-upper Callovian), Rosso Ammonitico Medio (RAM, upper Callovian-middle Oxfordian) and Rosso Ammonitico Superiore (RAS, middle Oxfordian-upper Tithonian), as formalized by Martire et al. [47] in the area of Asiago (Figs 1 and 2). The RAI is generally quite massive and calcareous with a typical pseudonodular facies. The RAM is characterized by thin-bedded, planar-parallel- to flaser-bedded limestones locally associated with nodules and layers of red chert while the RAS consists of pink-red nodular ammonite-rich limestones with brick-red clay-rich matrix [26, 47]. In the Feltre and Belluno areas (e.g., Ponte Serra; Figs 1 and 2), sandwiched between the RAI and RAS, crop out several tens of meters of thin bedded red and gray cherty limestones that also include resedimented ooidal-peloidal grainstones (e.g., [48]). These beds partly correlate to the RAM and correspond to the Fonzaso Formation, whose deposition probably took place in a fault-bounded, early drowned eastern block of the Trento Plateau [47]. The paleobathymetry of the RAV is much debated, with more recent studies suggesting a pelagic environment not excessively deep (from a deep photic zone to a few hundred meters [46, 47].

Finally, most of the fossils described herein derive from quarrying activity, with the RAV being used as decorative and building stone since Roman times (e.g., [49]).

## Material and methods

### Specimens

All necessary permits were obtained for the described study by the Soprintendenza Archeologia, Belle Arti e Paesaggio of Venezia and its Lagoon, the Chioggia Municipality, the Sistema Museale di Ateneo of Bologna University and by the Centro di Ateneo per i Musei of the Padova University, which complied with all relevant regulations. Nine specimens are included in this study (Table 1), here listed in order of discovery, including all relevant repository information.

1) MGP-PD 26552, complete mandible, portion of the skull roof, imprint of the anterior portion of the rostrum and teeth of a metriorhynchid found at Tresché (Asiago Plateau, Vicenza province; Fig 1) in 1787, and housed in the Museum of Nature and Humankind (MNH) of the University of Padova (formerly Museum of Geology and Paleontology). This specimen is the holotype of *Steneosaurus barettoni* (e.g., [27, 28, 36, 51]). The fossil, originally belonging to the Barettoni family of Schio (Vicenza), was acquired in 1936 by Giorgio Dal Piaz on behalf of the University of Padova.
2–3) MGP-PD 6761 and MGP-PD 6572, two isolated vertebrae from Cesuna (Asiago Plateau, Vicenza province; Fig 1) originally ascribed by De Zigno [27] along with other eight elements (vertebrae and neural arches) to a plesiosaurian (“Plesiosaurus italicus”, *nomen in schedis*; see also [53]). MGP-PD 6572 was recognized by Cau & Fanti [36] to be part of a chimerical association of crocodylomorph centrum and plesiosaurian neural arch. MGP-PD 6761 was mislabeled in Cau & Fanti [36] as MGP-PD 6571. Both specimens are housed in the collection of the MNH of the University of Padova.
4) The “Portomaggiore crocodile”, four polished slabs (here intended as a single specimen) with sectioned cranial and cervical vertebrae of the holotype of the metriorhynchid *Neptunidraco ammoniticus* [25]. Found in Portomaggiore (Ferrara province, Emilia Romagna) in 1955, the specimen is believed to come from S. Ambrogio di Valpolicella (Verona province; Fig 1). The specimen is housed in two different museums in two separate cities: Collezione di Geologia “Museo Giovanni Capellini’’ in Bologna (slabs MGGC 8846/1UCC123a and MGGC 8846/1UCC123b) and the Museo Paleontologico e della Preistoria Piero Leonardi (slabs MPPPL 35 and MPPPL 39) in Ferrara.
5) MM 25.5.1078, sectioned crocodylomorph dentigerous rami on a polished slab of reddish subnodular limestone coming from an unknown quarry of RAV in the Asiago Plateau (Vicenza province; Fig 1). It was found in 1966 and is housed at Museo Padre Aurelio Menin in Chiampo (Vicenza). This specimen, mislabeled as an “ichthyosaur” has not been previously described.
6) MGP-PD 27566, osteoderms, long bones, vertebrae, and pelvic elements of an aeolodontin teleosauroid [28, 34]. Found in 1980 at Ponte Serra (Belluno province; Fig 1). This specimen is housed in the MNH of the University of Padova.
7) MGP-PD 32438, skull roof, mandibles, vertebrae, ribs and one tooth of a metriorhynchid found in a quarry at Cima del Porco (Asiago Plateau, Vicenza province; Fig 1) by Francesco Massari in 1986. Previously listed as “ichthyosaur” [29], this specimen has not been so far described and is housed in the MNH of the University of Padova.
8) FOS03839, sectioned dentigerous rami of a metriorhynchid on six polished slabs found in 1990 at Valbella Quarry (Sasso d’Asiago, Vicenza province (Fig 1) [28, 31], housed in Museo di Scienze e Archeologia-Fondazione Museo Civico di Rovereto (FMCR, Trento).
9) MCLSC T2 (preliminary specimen number), articulated vertebral column, ribs, pelvic and hind limbs elements of a metriorhynchoid in a RAV boulder. The specimen was found in the 1990’s in one of the breakwater barriers in Pellestrina (Lagoon of Venezia) and is presently housed in the courtyard of the Museo Civico della Laguna Sud di Chioggia (Venezia). The quarry the boulder originated from is possibly Valbella Quarry at Sasso d’Asiago (Fig 1; Fabrizio Bizzarini, pers. com.) The specimen has not been previously described.

**Table 1:** Review of all thalattosuchians so far described from the RAV.

Catalog number	Composition	Taxonomy	Provenance	Age	Description status
MGP-PD 26552	Mandibles, imprints of anterior rostrum, incomplete skull roof	1890 *Steneosaurus barettoni*, Omboni; 2014 *Neptunidraco* sp., Cau; 2016 *Neptunidraco ammoniticus* Cau & Fanti; 2019 Metriorhynchoidea *incertae sedis*, Cau. This study: Metriorhynchidae indet.	Tresché Conca, Asiago Plateau (Vicenza province)	middle Oxfordian-early late Oxfordian	Described [36, 52], this study
MGP-PD 6761 + MGP-PD 6752	Isolated vertebra + chimerical isolated vertebra	*Plesiosaurus italicus*, De Zigno (*nomen in schedis*); 1883 *Plesiosaurus*, De Zigno; 1997 *Plesiosaurus italicus*, Dalla Vecchia; 2016? Thalattouschia indet., Cau & Fanti. This study: Sauropsida indet.	Cesuna, Asiago Plateau (Vicenza province)	latest Oxfordian-Kimmeridgian	Described [27, 35]
MGGC 8846/1UCC123a, MGGC 8846/1UCC123b, MPPPL 35, MPPPL 39	Incomplete and sectioned skull, mandibles and cervical vertebrae	1956 *Metriorhynchus*, Leonardi; 1980 *Metriorhynchus*, Kotsakis & Nicosia; 2009 *Geosaurus*, Young & Andrade; 2011 *Neptunidraco ammoniticus*, Cau & Fanti; 2019 Metriorhynchidae (*N*. *ammoniticus* emended), Cau	S. Ambrogio di Valpolicella (Verona province)	latest Bajocian-earliest Bathonian	Described [25, 31, 87]
MM 25.5.1078	Partial sectioned maxilla with teeth	This study: Metriorhynchoidea *incertae sedis*	Unknown quarry of Asiago Plateau (Vicenza province)	middle-late Bajocian	Undescribed until this study
MGP-PD 27566	Osteoderms, dorsal, sacral and caudal vertebrae, left ischium,? ribs-gastralia	1994 *Steneosaurus* sp., Sirna et al.; 1996 *Steneosaurus* sp. Bizzarini; 2022 Aeolodontinae, Serafini et al. This study: Aeolodontini	Ponte Serra (Belluno province)	latest Kimmeridgian- earliest Tithonian	Described [28, 34]
MGP-PD 32438	Incomplete skull roof, incomplete mandibles, single isolated tooth, cervical vertebrae, cervical ribs	This study: Metriorhynchidae indet.	Cima del Porco, Asiago Plateau (Vicenza province)	latest Bajocian-late Bathonian	Reported in [29]; undescribed until this study
FOS03839	Incomplete sectioned dentigerous elements	1996 Metriorhynchidae, Bizzarini; 2014 *Neptunidraco* sp., Cau; 2019 Metriorhynchoidea *incertae sedis*, Cau	Sasso d’Asiago (Vicenza province)	Bathonian?	Described [28, 35, 31]
MCLSC T2 (uncatalogued)	Partial column, ribs, femur, ischium, pubis	This study: Metriorhynchoidea *incertae sedis*	Unknown, possibly Sasso d’Asiago (Vicenza province)	middle-late Bajocian	Undescribed until this study

### Osteological and taphonomical analysis

The new specimens were personally measured by GF and GS with a digital caliper to the nearest millimeter; measurements focused on vertebral parameters (centrum height CH, centrum length CL; see S1 Table) and skull roof ratios between elements, such as width and distance of the postorbital from the prefrontal bones, angle between median and lateral process of the frontal (*sensu* [54]) and angles between prefrontal lateral margins and the axis of the frontal. Pictures were taken with either a Canon 700D or a Sony ILCE-7RM3. The 3D model of the prefrontals imprints and frontal-postorbital complex of MGP-PD 32438 a was produced using photogrammetry with the software Agistoft Photoscan. The topographical depth map of MGP-PD 26552 was created using the same software and technology. For most specimens, UV-induced fluorescence was used to discriminate anatomical, histological and taphonomical details on the skeletal tissue, creating a distinct contrast in color with the surrounding matrix. UV-A (peak emission at 368 nm), UV-B (peak emission at 318 nm) and UV-C (peak emission at 254 nm) wavelength were produced with a 95 W discharge lamp from WayTooCool LLC. For MCLSC T2 the detergent Algae.net (©Fila) was applied on the surface of the boulder to clean the skeletal elements from lichens and mosses built up during years of weathering which hampered the osteological characterization. A detailed tapho-morphological analysis was carried out for each specimen, gathering numerical data as a scoring for degree of articulation, completeness and erosion of the skeletal tissue (S2 Table). The scoring for articulation and completeness is specific for each anatomical district (head, anterior-posterior spine, right and left forelimb-hindlimb) modified after Beardmore et al. [55]. A custom percentage scoring for degree of compact tissue erosion was applied to non-sectioned/polished specimens. The description and taxonomic determination of the specimens follows a strict apomorphy-based approach [43, 44].

### Micropaleontological analysis

Small samples of matrix were extracted, when possible, from the specimen slabs to conduct micropaleontological analysis. To analyze the calcareous nannofossil content, samples were treated according to the smearing technique [56] for marly samples (e.g., MGP-PD 26552) or, alternatively, were processed according to the modified settling technique of Flores & Sierro [57] for more calcareous samples. Calcareous nannofossil assemblages were semi-quantitatively estimated by counting all the coccoliths and nannoliths recorded in 300 fields of view. Relative species abundances are reported as: A = abundant, at least 1 individual every 1–10 observation fields; C = common, 1 individual every 1–10 observation fields; F = frequent, 1 individual every 10–30 observation fields; R = rare, 1 individual every > 30 observation fields. Biostratigraphy is described with reference to the biozonation scheme of Casellato [58]. These analyses were performed using a polarized light microscope under a magnification of 1250x.

### Institutional abbreviations

MNH: Museo della Natura e dell’Uomo (Museum of Nature and Humankind), Padova University, Italy; MGP-PD: Museo di Geologia e Paleontologia di Padova (old MNH designation), presently Section of Geology and Paleontology of the MNH; MGGC: Collezioni di Geologia “Museo Giovanni Capellini”, Bologna, Italy; MPAMC: Museo Padre Aurelio Menin in Chiampo, Chiampo, Italy; MPPPL: Museo Paleontologico e della Preistoria Piero Leonardi of Ferrara, Ferrara, Italy; MCLSC: Museo Civico della Laguna Sud di Chioggia, Chioggia, Italy; FMCR: Fondazione Museo Civico di Rovereto, Rovereto, Italy; NHMUK: Natural History Museum, London, UK.

### Lithological abbreviations

RAV: Rosso Ammonitico Veronese; RAI: lower member of the Rosso Ammonitico Veronese (Rosso Ammonitico Inferiore); RAM: middle member of the Rosso Ammonitico Veronese (Rosso Ammonitico Medio); RAS: upper member of the Rosso Ammonitico Veronese (Rosso Ammonitico Superiore).

## Results

### MGP-PD 26552: Barettoni’s metriorhynchid

#### Systematic paleontology

Superorder: Crocodylomorpha Hay, 1930 [59]

Suborder: Thalattosuchia Fraas, 1901 [60] *sensu* [9]

Superfamily: Metriorhynchoidea Fitzinger, 1843 [61] *sensu* [9]

Family: Metriorhynchidae Fitzinger, 1843 [61] *sensu* [9]

“*Steneosaurus*” *barettoni* [36]

Figs 3–5

**Fig 3:**
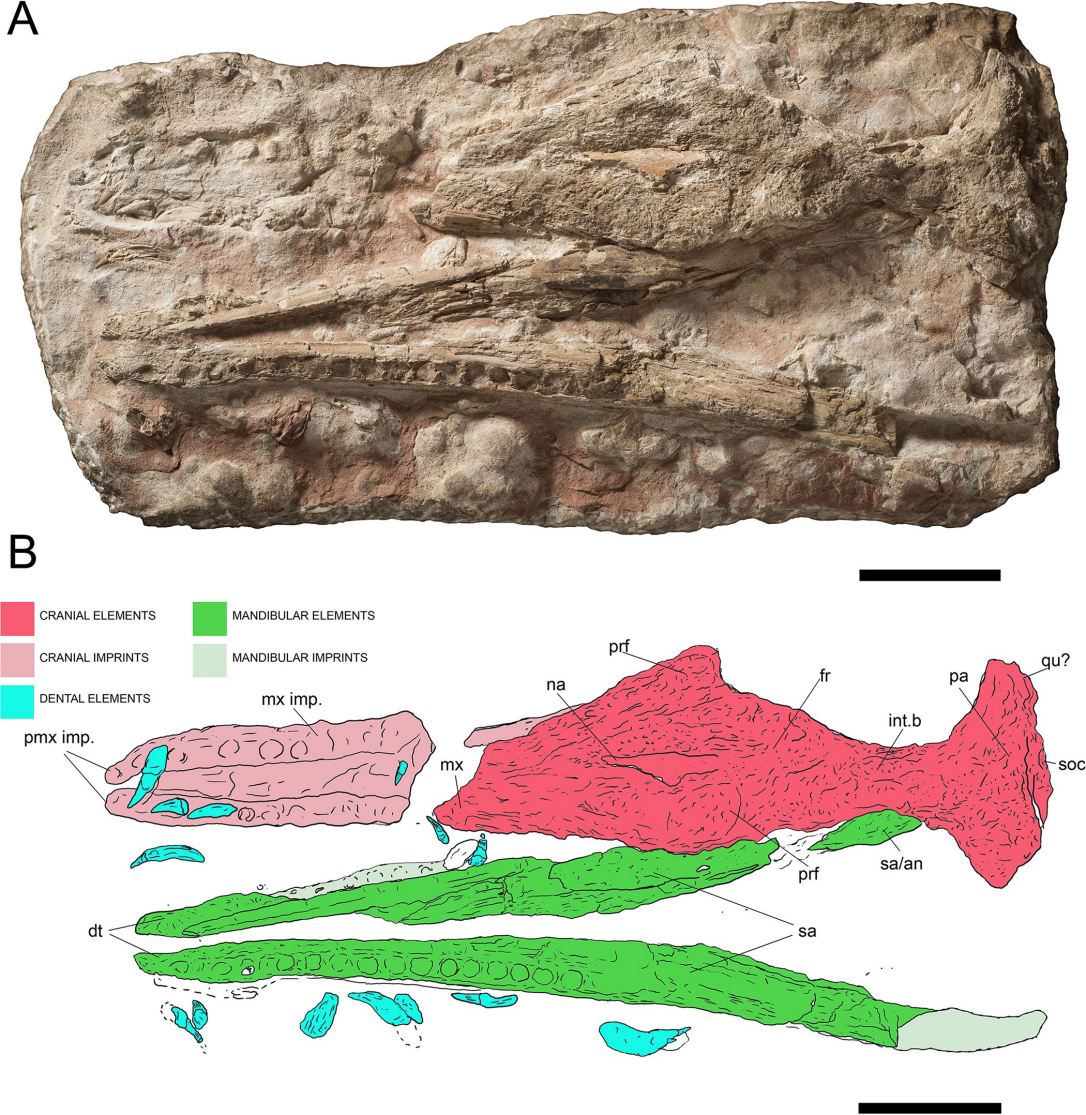
Overview of MGP-PD 26552, *“Steneosaurus” barettoni*. A) Orthogonal picture of the specimen. B) Anatomical drawing of the specimen with color-based differentiation of the main elements. Abbreviations: an, angular; dt, dentary; fr, frontal; int.b, infra-temporal bar, j, jugal; mx, maxilla; mx imp., maxillary imprint; na, nasal; pa, parietal; pmx, premaxilla; pmx imp., premaxillary imprint; prf, prefrontal; qu, quadrate; sa, surangular; soc, supraoccipital. Scale bars represent 10 cm.

**Fig 4:**
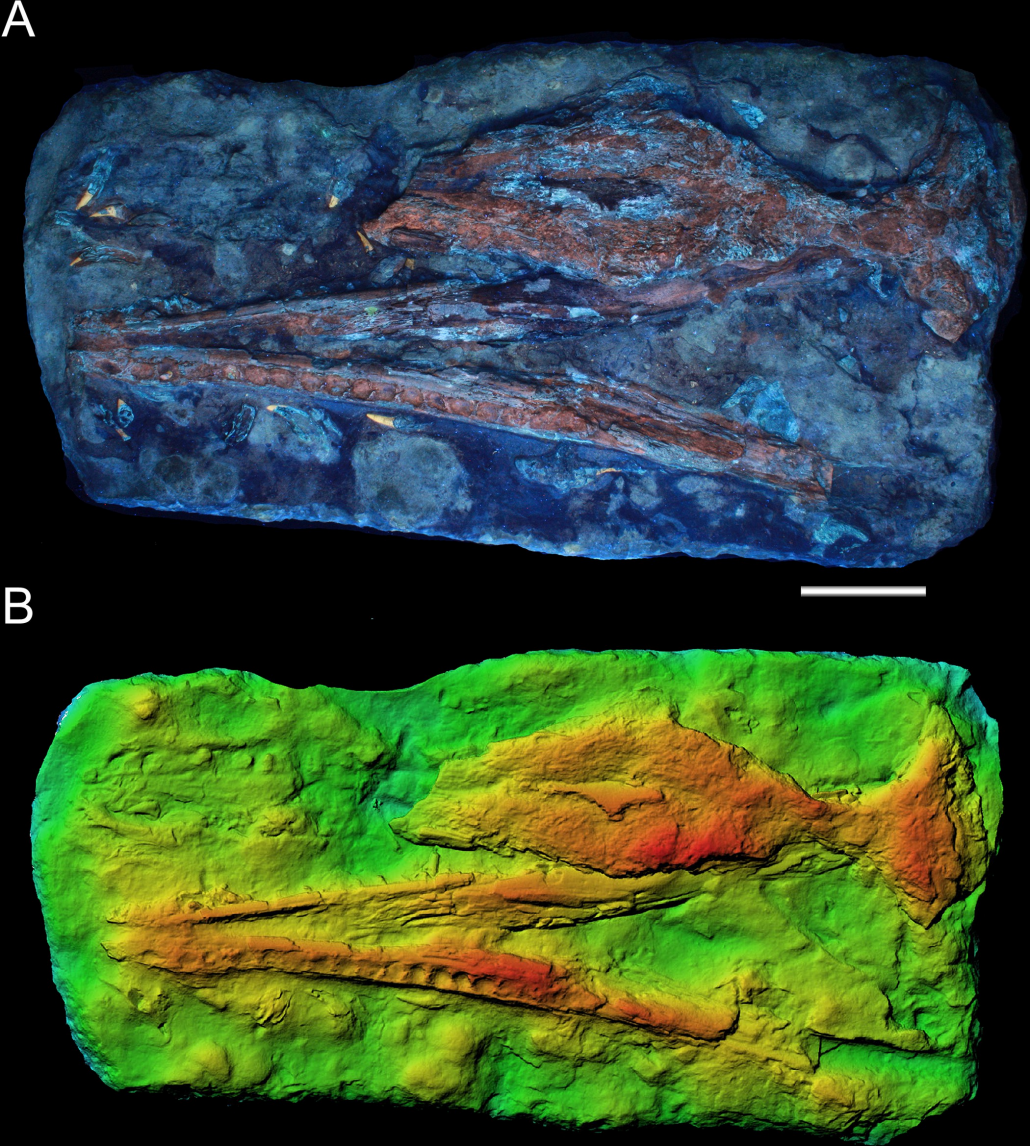
Surface analysis of MGP-PD 26552. A) MGP-PD 26552 under ultraviolet radiation (UVA-UVC). Bright yellow shows the tooth enamel response, orange-red shows the cancellous bone response. B) Depth map of MGP-PD 26552 surface by means of photogrammetry. Warmer colors indicate more elevated regions. Scale bars represent 10 cm.

**Fig 5:**
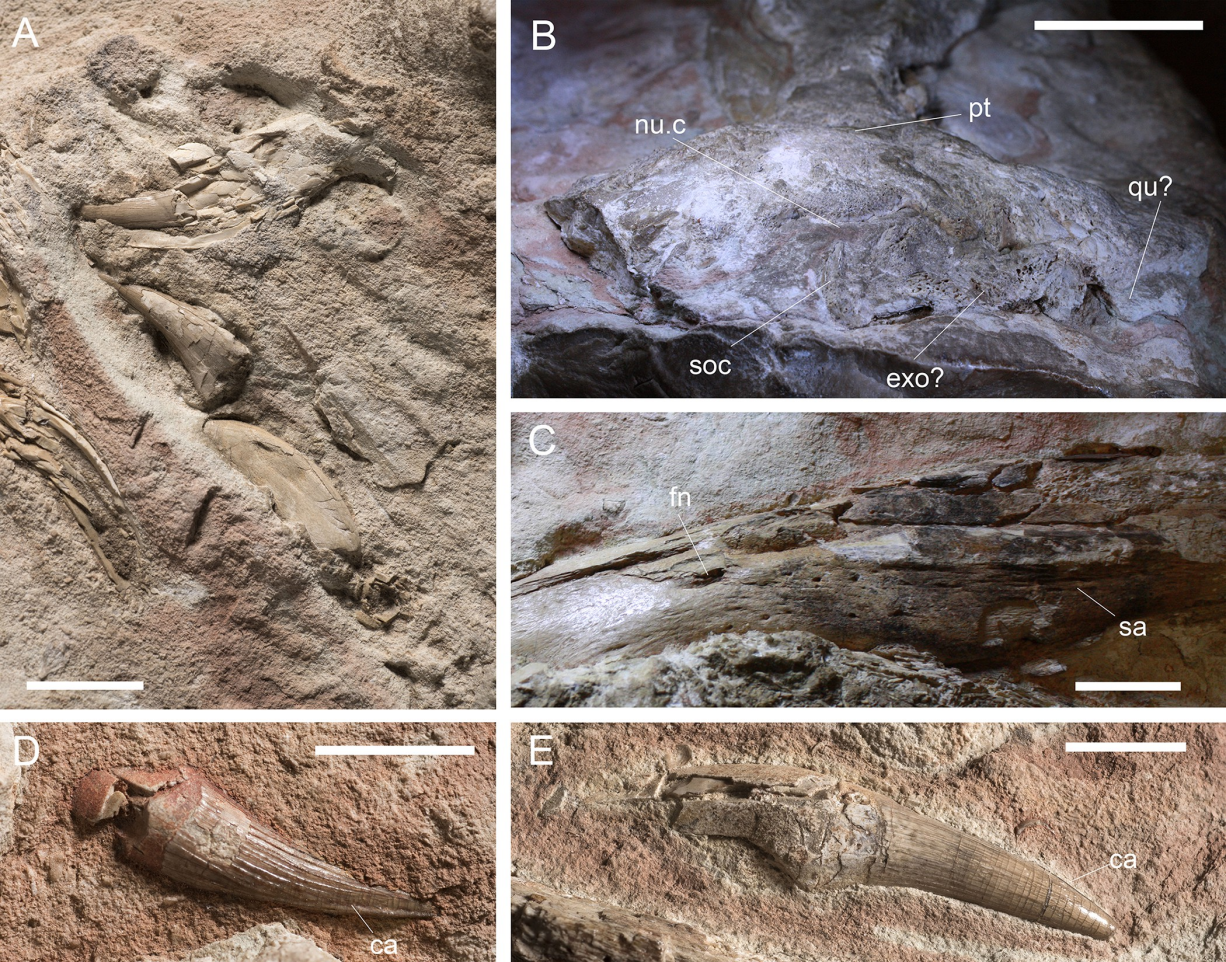
MGP-PD 26552 details. A) Close-up of the premaxillary imprint with corresponding dislodged teeth. B) Detail of the occipital region of the skull. C) Close-up of the best-preserved mandibular ramus showing 3D foramina. D) Close-up of a posterior tooth in labial view. E) Close-up of a posterior tooth in lingual view. Abbreviations: ca, carina; exo, exoccipital; fn, foramina, int.bar, intertemporal bar; nuc., nuchal crest; pa, parietal; qu, quadrate; soc, supraoccipital; Scale bars: A) 2 cm; B-C) 3 cm; D-E) 1 cm.

Locality and horizon: Tresché Conca (Asiago Plateau, Vicenza province, northern Italy: Fig 1). Historically attributed to the Tithonian beds of the Rosso Ammonitico Veronese (i.e. RAS; see [28, 53]). Here considered to be middle-upper Oxfordian (see below).

#### Age reassessment and stratigraphic provenance

A small sample of brick-red marl has been removed from the side of the slab preserving the fossil for preparation of a smear slide. The calcareous nannofossil assemblage of MGP-PD 26552 presents the following composition: *Watznaueria* aff. *manivitae* (C), *Watznaueria manivitae* (F), *Watznaueria communis* (C), *Watznaueria britannica* (F), *Watznaueria barnesiae* (R); *Cyclagelosphaera margerelii* (R), *Schizosphaerella puctulata* (fragments), *Lotharingius hauffii* (R). Fragments similar to *Faviconus*. *multicolumnatus* are actually fragments of the fibrous crust of *S*. *punctulata*. For the presence of *Lotharingius hauffii* in the absence of *Lotharingius sigillatus* and *F*. *multicolumnatus*, the sample is attributable to the NJT13b subzone [58] and therefore MGP-PD 26552 can be ascribed to the middle Oxfordian (p.p.)—base of upper Oxfordian (Fig 2A). Despite the biostratigraphic dating, the lithostratigraphic assignment of the fossil is problematic, considering that our study is not supported by microfacies analysis (a removal of a limestone fragment from the slab was not allowed). At Asiago, the middle Oxfordian is usually recorded at the top of RAM in subnodular white to light pink cherty limestones that yielded ammonites indicating the middle Oxfordian *Gregoryceras transversarium* Zone, whereas the upper Oxfordian is not documented [26, 47]. The lithology of MGP-PD 26552, however, is a reddish nodular micritic limestone with thin red brick marly interbeds more reminiscent of the typical RAS facies. We tentatively assign MGP-PD 26552 to the base of RAS, in spite of the fact that in the Asiago area the middle Oxfordian *Gregoriceras transversarium* Zone has been so far recorded in the RAS only at Echar section [26] and is represented by a stromatolitic facies, not observed on the matrix of the study fossil. Considering the extreme lateral variability of thickness and facies of the RAV, it is possible that at Treschè was exposed a so far unknown “anomalous” succession with a differently expressed facies of the mid Oxfordian interval and/or with a previously unknown record of the upper Oxfordian (Fig 2A).

#### Taphonomy and preservation

The preservation of MGP-PD 26552 is not optimal. The anterior half of the rostrum is missing, along with the lateral side of the supratemporal fenestra, and posterior lower jaws (Fig 3A and 3B). The specimen suffered a strong dorsoventral compression of the cranium, and mandibles. While the completeness of the skull and mandible is poor, articulation is relatively high, with at least 11 elements still close to the anatomical connection. From a histological perspective, MGP-PD 26552 is characterized for the most part by exposed cancellous bone, often with unrecognizable trabecular structure due to compression and dissolution (Fig 3A). The erosion of the skull roof elements prevents a precise assessment of the sutures. As highlighted by UV induced fluorescence (Fig 4A), the percentage of exposed cancellous bone stands for more than 60% of the entire specimen. Dentine and enamel, when preserved, instead suffered minor superficial damage. The skull roof appears to have experienced most of the surface erosion, and most of the compact tissue preserved on the specimen occurs in the lower jaw. The mandibles of MGP-PD 26552 might have been shielded by the upper portion of the skull during decay, possibly facilitating and hastening its burial. Eight rhyncholites were identified around MGP-PD 26552, while a single *Lamellaptychus* was found between anterior teeth in the left premaxilla-maxilla imprint, possibly associated with early stages of the specimen deadfall ecology ([62]; GS preliminary pers. observ.).

#### Description

We largely agree with the anatomical interpretation by Cau [52], and we will use this section to report new data or differences from previous works. MGP-PD 26552 (catalog erroneously spelled as 6552 in [31, 35, 52, 63]) is composed by almost complete mandibles still in articulation with teeth dislodged from the sockets, by remnants of the mid-posterior portion of the skull roof and by an imprint of the anterior portion of the rostrum with related dislodged teeth (Fig 3A and 3B). Cranial remains are preserved in a roughly 1 x 0.5 m slab of highly nodular RAV limestone. The anterior imprint of the rostrum represents the dentigerous premaxilla and maxilla, with nine recognizable alveoli for the right ramus and eight for the left one. As in all other metriorhynchids (e.g., [2, 64]) each premaxilla has 3 alveoli (Fig 5A); a small diastema, well-visible on the right side separates the final premaxillary alveoli from the first maxillary alveoli. The mid portion of the rostrum is dorsoventrally flattened, the nasals are eroded and only a fragment of the left maxilla is preserved (Fig 3A and 3B). The nasals expand posteriorly, forming a slight depression along the posterior rostrum midline [52]. A small fragment of the left nasal preserves compact tissue that did not collapse, overall representing the best-preserved element from the specimen skull roof (Fig 3A). The ornamentation on the external surface of the nasals is not evident. Cau [52] recognized the right prefrontal, positioned lateral to the right nasal bone, close to the right edge of the slab (see Figs 1B and 2B in [52]). We additionally identified the shape of the left prefrontal between the cancellous surface of the left nasal (Fig 3B): this element is more elevated than its right counterpart, with distinct teardrop shape; its complex surface texture is likely due to underlying trabecular structures rather than surface ornamentations. The sutures between the nasal, prefrontal and frontal cannot be confidently recognized due to the strong erosion and dorsoventral deformation. The postorbitals are missing and the shape of supraorbital notches (in dorsal view) cannot be confidently assessed due to collapsed elements and matrix. The intertemporal (= frontoparietal) bar is robust. The posterior end of the skull roof is represented by the supraoccipital and possibly by quadrate fragments in a “fan-like” single structure (Fig 5B). The supraoccipital appears dorsally concave, and possibly accommodated a nuchal crest in the complete and undeformed skull. Small portions of the exoccipitals might also be present (Fig 5B). Our interpretation of the mandibles mostly follows that of Cau [52], with same socket count (i.e., ~17 on the left ramus), absence of reception pits for premaxillary teeth in the anteriormost dentary, posterodorsal projection of the dentary, extensive splenial contribution to the mandibular symphysis, angular contribution and the lack of preserved retroarticular processes (Figs 3A, 4A, 4B and 5C). We find it difficult to understand the full extent of the mandibular symphysis, but we do not consider it to be as shortened as [52] interpreted. Seventeen loose tooth crowns can be identified on the slab, especially under UV light (Fig 4B). The anterior teeth have long roots and small curved crowns (circa 2 cm in apicobasal length), with apicobasal enamel ridges that are few and discontinuous (Fig 5A). Some of the posterior teeth had much longer crowns (Fig 5D and 5E), with a less distinctive apicobasal curvature than the anterior ones and much more coarse enamel ridges [31, 52]. The carinae are partially visible in only two crowns (Fig 5D, 5E), there is no evidence of false serrations (enamel ornamentation contacting the carinae) and true denticles were not identified.

#### Taxonomy

Despite the early discovery in the late 18th century and a long history in the literature (e.g., [65–74], among others) MGP-PD 26552 was named by Baron Achille De Zigno only in 1883 as *Steneosaurus Barettoni* and was later described in detail by Omboni [36]. MGP-PD 26552 is the holotype for *Steneosaurus barettoni*, a taxon that was considered valid by Steel [75], Bizzarini [28], Dalla Vecchia [63], and Delfino & Dal Sasso [37]. Bizzarini [28] questioned, for the first time, the validity of the attribution to *Steneosaurus*, recognizing the length of the mandibular symphysis of MGP-PD 26552 to be inconsistent with *Steneosaurus* and Teleosauridae (although note in the 19th Century there was a time when *Steneosaurus* was used as the senior synonym of *Metriorhynchus*, see [64, 76]). The first in-depth and modern revision of the specimen by Cau [52] considered *Steneosaurus barettoni* to be a *nomen nudum* because the establishment of the nomen by De Zigno [27] failed to conform to Article 12 of the International Code of Zoological Nomenclature [77]. Cau [52] instead assigned the “unnamed” specimen to Metriorhynchidae and to *Neptunidraco* sp., a genus erected by Cau & Fanti [25]. This classification was followed by Cau & Fanti [35], who inferred that the lineage leading to *Neptunidraco* had undergone unusually high rates of divergence from other metriorhynchid taxa. Later Cau [31] tested his previous phylogenetic hypothesis with a Bayesian specimen-level methodology (Fossilized Birth-Death Sampled Ancestor model) and following an anatomical revision, MGP-PD 26552 was re-interpreted as Metriorhynchoidea *incertae sedis*.

The genus *Steneosaurus*, a historical waste-basket genus for Lower to Upper Jurassic longirostrine teleosauroids [10, 78], was considered a *nomen dubium* by Johnson et al. [76]. Unambiguously diagnostic characters on MGP-PD 26552 are scarce, although it has an extensive participation of the prefrontal in the orbit anterior margin (Metriorhynchoidea apomorphy, [64]), the prefrontal dorsal surface is greatly expanded laterally, overhanging part of the orbit (*Zoneait* + Metriorhynchidae apomorphy: [64]) and a true supraorbital notch can be recognized (Metriorhynchidae apomorphy: [64]). Moreover, broad and short, posteromedially oriented and roughly tear-drop shaped prefrontals represent a clear metriorhynchid feature [9, 64]. A frontal wider than 75% of the parietal was considered to be a metriorhynchid apomorphy by Young & Andrade [9], but in MGP-PD 26552 the dorsoventral merging of multiple cranial elements hampers accurate measurements. We identify the following Geosaurinae apomorphies: cranial rostrum with a mesorostrine condition created by elongation of the maxilla (shared with *‘Cricosaurus’ saltillensis*; [64]), and 20 or fewer maxillary alveoli (within Metriorhynchidae, shared with the Cretaceous rhacheosaurin: [64]). It is noteworthy that the nuchal crest potentially present in MGP-PD 26552 (Fig 5B) is shared also by the geosaurine *“Metriorhynchus” brachyrhynchus* [79]. The Oxfordian age of the specimen is inconsistent with any non-metriorhynchid metriorhynchoid taxa (e.g., [3, 14–16, 80, 81]) as early-diverging metriorhynchoids are only known from Lower-Middle Jurassic deposits; furthermore, no metriorhynchoid more derived than *Magyarosuchus* has an external mandibular fenestra (which is also absent in MGP-PD 26552; MTY preliminary observations). Due to the new age determination, the presence of three premaxillary alveoli, laterally expanded prefrontal, we conservatively assign MGP-PD 26552 to Metriorhynchidae indet. The specimen shares some features with Geosaurinae, but with the available material we opted for a more conservative approach.

#### Nomenclatural issues

Cau [52] stated that *Steneosaurus barettoni* in De Zigno [27] is a *nomen nudum* as its establishment did not conform to Article 12 of the ICZN Code [77], due to it being “accompanied by a description or a definition of the taxon it denotes”. This is correct, because De Zigno ([27], p. 322) indeed did not provide any description or illustration of this specimen. However, when Omboni [36] described and figured the fossil, he treated the name coined seven years before by De Zigno (“*Steneosaurus Barettoni* Zigno”) as a valid synonym. Therefore, under Articles 11 and 12 of the ICZN Code [77] the specific epithet *barettoni* is indeed available. As *nomina nuda* are not available names, the same nomen may be made available later for the same or a different concept. In such a case, authorship (Arts. 50, 21) comes from the act of establishment, not earlier publications as a *nomen nudum* ([77]; see online glossary https://code.iczn.org). Therefore, the specific epithet *S*. *barettoni* is available and was established by Omboni [36]. Herein we propose to continue to refer MGP-PD 26552 to "*Steneosaurus*" *barettoni*. The use of open nomenclature signs as quotation marks around the genus name clearly indicates, according to Bengtson [82], that the original generic assignment is obsolete in the context of systematic interest.

### MGP-PD 6761 and MGP-PD 6752: The Cesuna vertebrae

#### Systematic paleontology

Sauropsida indet.

Fig 6

**Fig 6:**
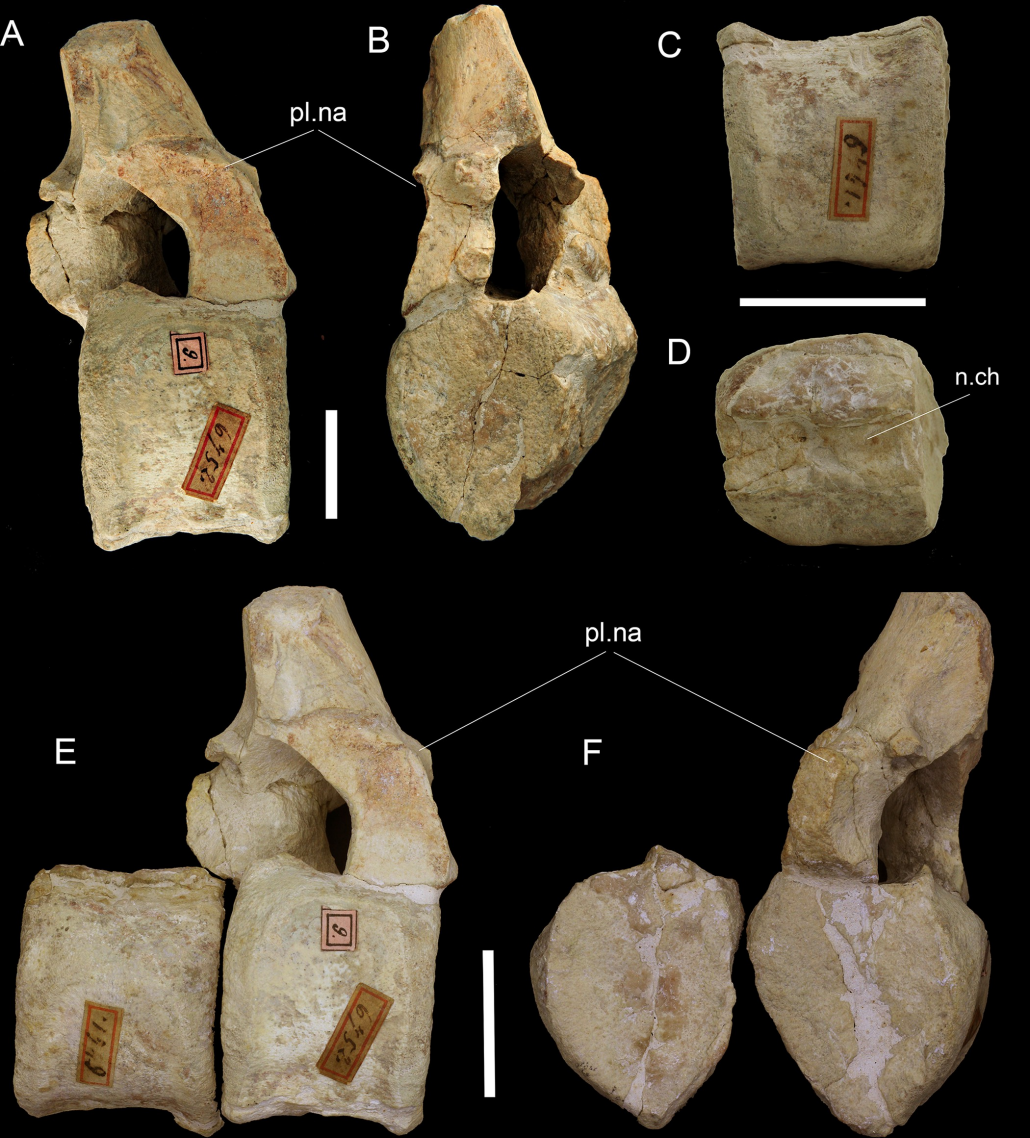
Vertebrae from Cesuna. A) MGP-PD 6572 in lateral view; B) MGP-PD 6572 in anterior view; C) MGP-PD 6761 in lateral view; D) MGP-PD 6761 in dorsal view; E) MGP-PD 6761 and MGP-PD 6572 in articulation; F) MGP-PD 6761 in anterior view and MGP-PD 6572 in posterior view highlighting the compatible matrix encrustation of the articular facets. Abbreviations: n.ch, neural canal; pl.na, plesiosaur neural arch. Scale bars represent: A-B) 2 cm; C-F) 3 cm.

Locality and horizon: Cesuna (Asiago Plateau, Vicenza province, northern Italy: Fig 1), Tithonian, upper unit of RAV (RAS), according to De Zigno [27] and his annotations on the original labels of the fossils (see also [35, 63]).

#### Age reassessment and stratigraphic provenance

The artificially composite MGP-PD 6752 does not retain traces of matrix, whereas a few occur in MGP-PD 6761, consisting of a pinkish limestone with abundant spatic calcite (possibly referable to echinoderm elements with syntaxial overgrowth). This sample is barren in calcareous nannofossils. More abundant matrix is still associated to the two vertebral plesiosaurian centra MGP-PD 6757 and 6759 (likely coming from the same outcrop; De Zigno [27]) and consists of a pinkish limestone resembling that of MGP-PD 6761 but differing for the presence of thin layers of red-brick marl. Of the two plesiosaurian vertebrae, MGP-PD 6757 was sampled. The calcareous nannofossil assemblage is poorly diversified and preserved and is characterized by the presence of: *Watznaueria* aff. *W*. *manivitae* (C), *W*. *manivitae* (F), *W*. *communis* (F), *W*. *barnesiae* (R), *Cyclagelosphaera margerelii* (R), *Faviconus multicolumnatus* (one fragment). MGP-PD 6757, based on the presence *F*. *multicolumnatus* and the absence of *Zeughrabdothus*, is ascribable to the lower-middle part of the NJT 14 zone of Casellato [58] corresponding to the uppermost Oxfordian-Kimmeridgian. If the alleged crocodylomorph and plesiosaurian vertebral centra of Cesuna come from the same horizon, they are confidently ascribable to the lower-middle portion of the RAS (Fig 2A).

#### Taphonomy and preservation

In addition to the Cau & Fanti [35] description, we note that the two preserved vertebrae are adjacent centra: the two elements are consistent in size (Fig 6A–6D), the two ventral keels are perfectly aligned (Fig 6E) and the intervertebral casts of sediments are complementary one with the other (Fig 6F). Because of this interpretation, the two vertebrae can be assigned to the same individual. Except for moderate axial deformations and lateral abrasions by weathering, both vertebral elements (together with the remaining plesiosaurian axial material from Cesuna; [35]) are in a good state of preservation, being three-dimensional and almost fully non-eroded. This type of preservation is not common for the RAV taphonomic regime; however, it has also been reported in an ichthyosaur rostrum fragment [53, 83] from the uppermost unit of the RAV of the Lessini Mountains. Subparallel scratches can be observed on the right dorsal edge of MGP-PD 6752 centrum, possibly representing traces of a shark bite.

#### Taxonomy

Cau & Fanti [35] tentatively assigned MGP-PD 6761 and MGP-PD 6752 to Thalattosuchia thanks to the exclusion of Plesiosauria (absence of ventral foramina, presence of a keeled pleurocentrum; [35]) and Ichthyopterygia (absence of notochordal pit/amphicoelic shape) and recognizing an overall closer resemblance of the two centra with crocodylomorph elements. Due to the strong lateral compression and abrasion, we cannot advance a reliable attribution to any group. Moreover, while ichthyosaurs can be feasibly excluded, we cannot rule out that the two centra might be plesiosaurian in nature. The absence of ventral foramina (*foramina subcentralia*) is an equivocal feature in Plesiosauria [84], as it is considered an apomorphy of the group only in cervical centra [85] and such foramina are greatly reduced in Pliosauridae [84, 86]. The moderate deformation of MGP-PD 6761 and MGP-PD 6752 might also be responsible for the *foramina subcentralia* not being recognized, together with their peculiar subtriangular shape in the articular view. Similarly, the presence of chevron facets or hypapophysis could have been obscured by lateral and ventral abrasions. The two centra could resemble flexural elements of metriorhynchids [21, 22], but with such limited available material we prefer to maintain a conservative and cautious approach. With a general attribution to Sauropsida indet. we could also account for the possibility of the two centra to belong to a continental taxon adrift at sea.

### MGGC 8846/1UCC123a-b, MPPPL 35–39: *Neptunidraco ammoniticus* holotype

#### Systematic paleontology

Superorder: Crocodylomorpha Hay, 1930 [59]

Suborder: Thalattosuchia Fraas, 1901 [60] *sensu* [9]

Superfamily: Metriorhynchoidea Fitzinger, 1843 [61] *sensu* [9]

Family: Metriorhynchidae Fitzinger, 1843 [61] *sensu* [9]

*Neptunidraco ammoniticus* [25]

Fig 7

**Fig 7:**
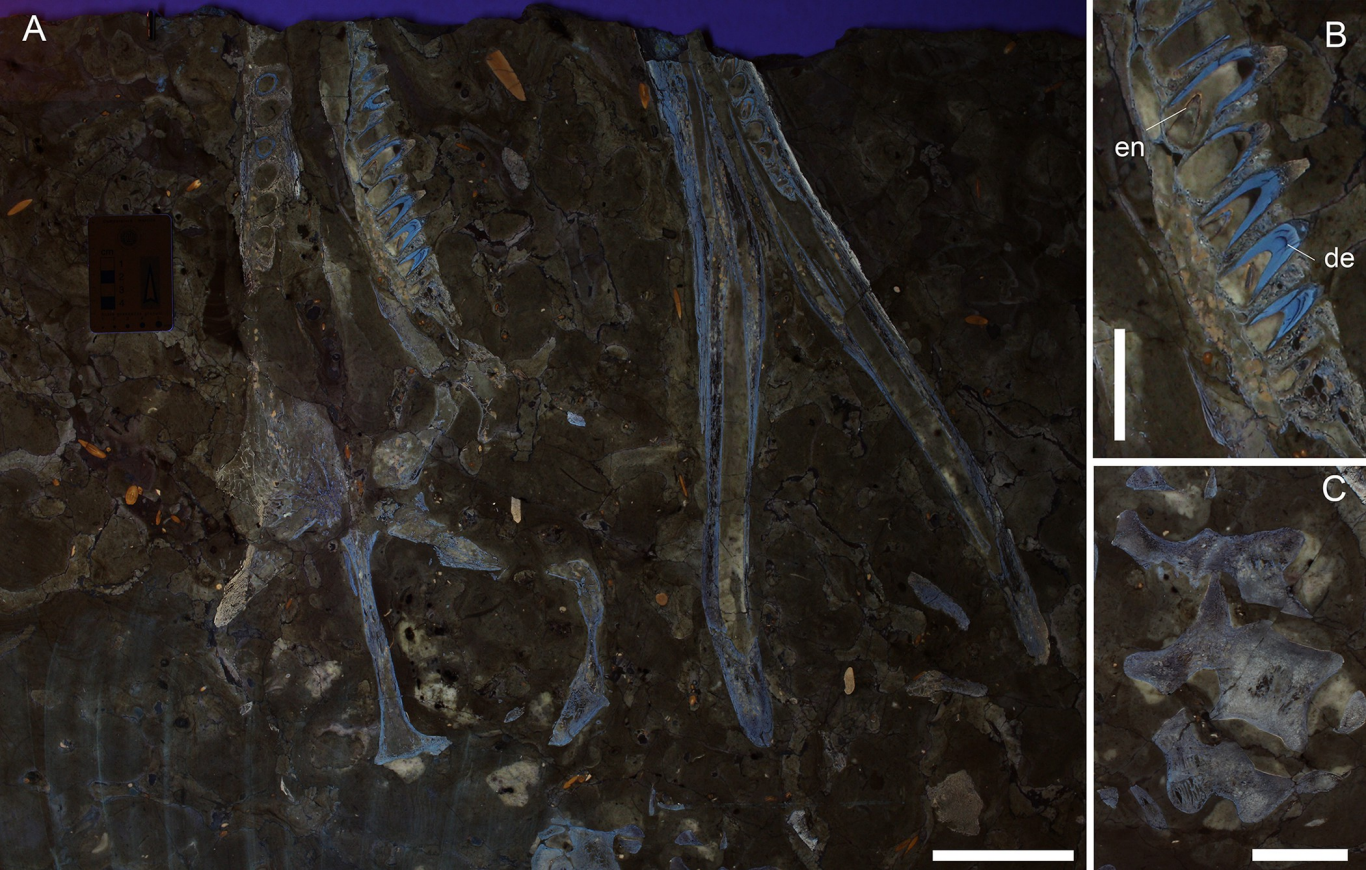
MGGC 8846/1UCC123a, holotype of *Neptunidraco ammoniticus* under ultraviolet light (UVA-UVB-UVC). A) Overview of the specimen serial section. B) Close-up of maxillary teeth showing the light lue response light of dentine under UV and the orange-yellow enamel response found only in the replacement crowns inside the corresponding roots. C) Detail of the cervical sectioned cervical vertebrae. Scale bars represent A) 10 cm; B-C) 5 cm.

Locality and horizon: Most likely from S. Ambrogio di Valpolicella (Verona province, northern Italy: Fig 1), upper Bajocian-lower Bathonian, RAV lower unit (RAI) according to Cau & Fanti [25].

#### Age calibration and stratigraphic provenance

Cau & Fanti [25] ascribed the specimen to the upper Bajocian-lower Bathonian thanks to petrographic and micropaleontological analysis. The microfacies of the slab matrix was interpreted as characteristic of the lower unit of the RAV (upper Bajocian- lower Callovian); the age of the specimen was then restricted to the upper Bajocian-lower Bathonian interval by means of planktonic foraminifera [25]. In this study we refer to this biostratigraphic assignment (Fig 2A).

#### Taphonomy and preservation

MGGC 8846/1UCC123a, MGGC 8846/1UCC123b, MPPPL 35 and MPPPL 39 contain portions of the skull, lower jaw and cervical vertebrae of a metriorhynchid in eight serial longitudinal sections (two each slab; [25]). The specimen is preserved on four polished slabs of compact RAV limestone from the lower member of the unit (RAI, Bajocian-Callovian; [26]). We largely agree with the anatomical interpretation of the specimen by Cau & Fanti [25]; part of the specimen (MGGC 8846/1UCC123a) is in permanent exhibition at the Collezione di Geologia “Museo Giovanni Capellini” and another at Museo Paleontologico e della Preistoria Piero Leonardi (MPPPL 35), so we were unable to access both side of these slabs (opposite side than the one on display). Because of this, any accurate osteological revision would currently be incomplete, and is beyond the scope of this study. Here we report novel data on the taphonomy and new information obtained from UV analysis (Fig 7). As already reported in Cau & Fanti [25], the specimen shows a very limited degree of cranial disarticulation (S2 Table), together with what appears to be a fair completeness of the skull as inferred by the longitudinal serial sections (Fig 7A). As for other RAV specimens preserved in polished slabs, superficial erosion of the skeletal tissue cannot be evaluated. No evident borings in the sectioned bone can be identified (Fig 7B and 7C). The specimen is histologically badly preserved: dental tissues are best preserved in the roots, where dentine is histologically recognizable and traceable by its UV-induced pale blue color (Fig 7A and 7B). Newly erupting crowns inside the roots are the only dental elements where enamel is preserved (thin outer layer with yellow UV-response light; Fig 7B), as primary crowns (outside the sockets) also appear highly eroded. Dentine is also absent in primary crowns, suggesting sediment substitutions in the tooth subsequent to water dissolution of the hard tissues. As also highlighted with the UV analysis, small (millimetric) subcircular objects can frequently be observed within the mandibular rami (Fig 7A). Although most-likely carbonatic in composition (due to the response light under UV typical of calcite) the nature of these structures remains unknown. Thanks to UV-induced fluorescence, more than 40 belemnite rostra can be identified around the skull in a vivid orange response light (Fig 7A). Many of the rostral fragments are found closely associated with the bones, with 5 individuals embedded between dorsal bones of the mid-skull roof (prefrontals-frontal contact and nasals). Sectioned belemnites display no preferential orientation, with equally recognizable cross-sectioned and longitudinal cuts.

#### Taxonomy

The taxonomic status of MGGC 8846/1UCC123a, b, MPPPL 35 and 39 underwent several changes: after its discovery in 1955 and recognition of crocodilian features (from thereon referred as the “Coccodrillo di Portomaggiore”–“The Portomaggiore Crocodile”), the specimen was firstly recognized as a metriorhynchid by Leonardi [87] and classified as a species of *Metriorhynchus*. This interpretation persisted in Kotzakis & Nicosia [88], until Young & Andrade [9] hypothesized close affinities to *Geosaurus*. Later, in a dedicated study, Cau& Fanti [25] found the “Coccodrillo di Portomaggiore” to display enough autapomorphies for the erection of a new genus and species, *Neptunidraco ammoniticus* (Geosaurinae), for which MGGC 8846/1UCC123a, b, MPPPL 35 and 39 become the holotype. Alongside MG-PD 26552 (“*Steneosaurus*” *barettoni* see above), the holotype of *N*. *ammoniticus* was considered to be a member of a rapidly divergent evolutionary branch of Metriorhynchidae in Cau & Fanti [35]. However, Cau [31] later considered the holotype of *N*. *ammoniticus* to be Metriorhynchidae *incertae sedis* (in the same study where the “*Steneosaurus*” *barettoni* holotype was considered Metriorhynchoidea *incertae sedis*). However, at least at present, *Neptunidraco ammoniticus* remains a valid taxon with a list of apomorphic characters [25]. Given our survey of only part of the holotype material, we cannot add anything to the taxonomic status of the specimen. Future work examining all sides of the holotypic blocks, (including the reverse side of display specimens), will be necessary.

### MM 25.5.1078: Unpublished specimen from Asiago Plateau

#### Systematic paleontology

Superorder: Crocodylomorpha Hay, 1930 [59]

Suborder: Thalattosuchia Fraas, 1901 [60] *sensu* [9]

Superfamily: Metriorhynchoidea Fitzinger, 1843 [61] *sensu* [9]

Metriorhynchoidea *incertae sedis*.

Figs 8 and 9

**Fig 8:**
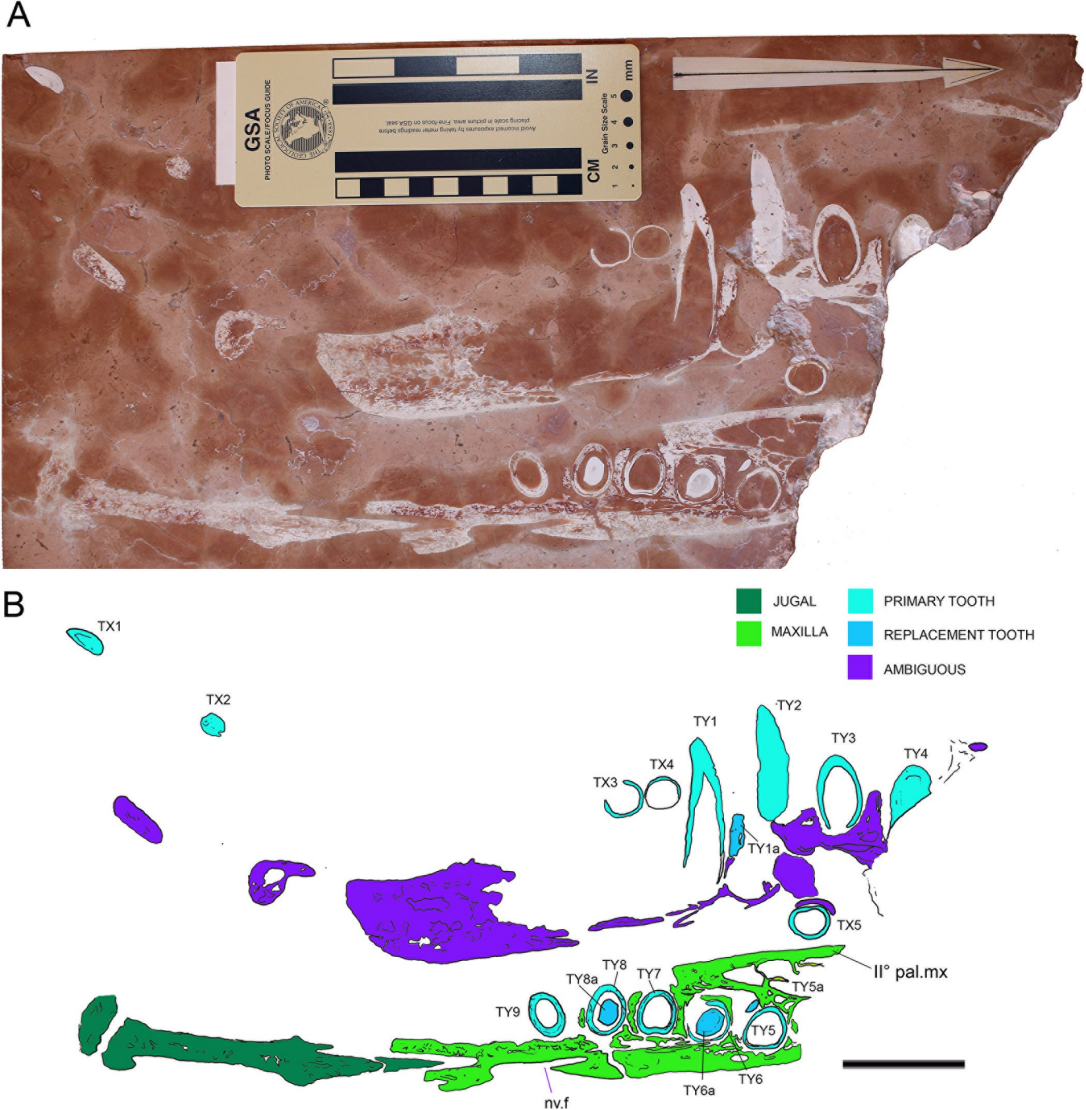
Overview of MM 25.5.1078. A) Orthogonal picture of the specimen. B) Anatomical drawing of the specimen with color-based differentiation of the main elements. Abbreviations: nv.f, neurovascular foramen; II° pal. mx, secondary palate maxilla, T, tooth. See text for teeth numeration. Scale bars represent 5 cm.

**Fig 9:**
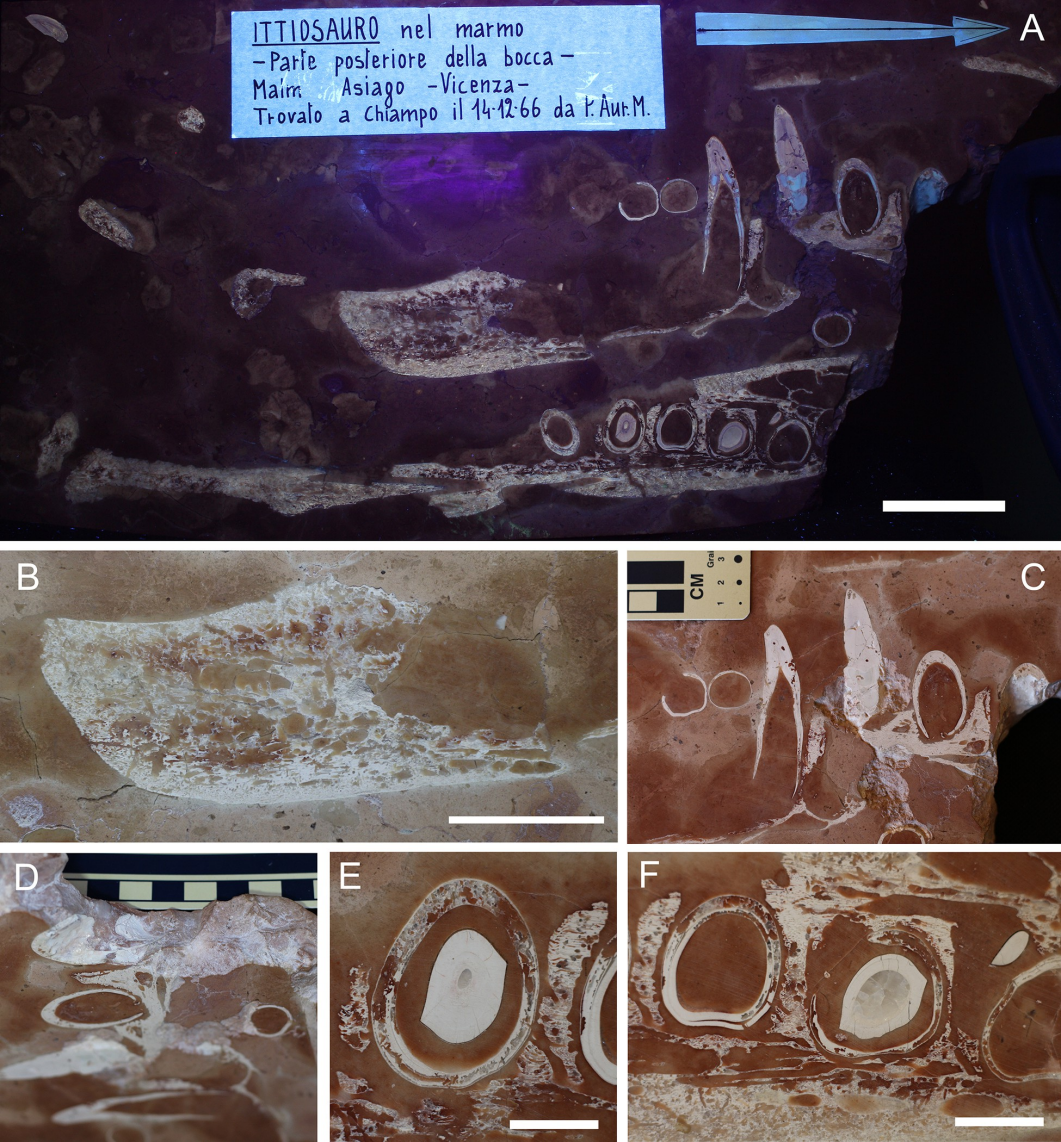
MM 25.5.1078 details. A) The specimen under ultraviolet radiation (UVA). The labels translate to “Ichthyosaur in marble—Posterior portion of the mouth—Malm Asiago, Vicenza—Found in Chiampo in 14/12/1966 by Father Aurelio Menin”. B) Detail of the eroded posterior portion of the upper dentigerous element. C) Teeth in longitudinal section from the upper dentigerous element. D) Cross section of the slab showing its thickness and the persistence of the bone-dental elements. E) Cross section of TY8 and its replacement. F) Cross section of (from the left) TY7 and TY6 and TY5 with corresponding replacement crowns/buds. Scale bars represent: A) 5 cm; B) 3 cm; E-F) 1 cm.

Locality and horizon: Unidentified quarry in the Asiago Plateau (northern Italy: Fig 1). Age and horizon of provenance originally unknown. Herein considered to be middle-upper Bajocian (see below).

#### Age calibration and stratigraphic provenance

Despite the cohesive nature of MM 25.5.1078 slab, a thin vein of marl on the unpolished side of the slab provided enough loose material for calcareous nannofossil analysis. Calcareous nannofossils are abundant and well preserved, consisting of *Watznaueria* aff. *W*. *manivitae* (C), *Watznaueria communis* (C), *Watznaueria manivitae* (C), *Watznaueria britannica* large (F), *Watznaueria gaetanii* (F), *Cyclagelosphaera margerelii* (R), *Lotharingius velatus* (R), *L*. *hauffii* (R), *L*. *sigillatus* (R), *Diazomatolithus lehmanii* (R), *Schizosphaerella punctulata* (R). For the presence of *W*. *manivitae* in the absence of *Carinolithus superbus* and *Watznaueria barnesiae*, the association can be referred to the subzone NJT10b [89]; therefore MM 25.5.1078 can be confidently assigned to the middle-upper Bajocian (see [90]). The age assignment and lithology of the slab allow to firmly assign the specimen to the lower portion of the RAI (Fig 2A).

#### Taphonomy and preservation

Despite the proximity of the two dentigerous rami, possibly close to anatomical articulation (Fig 8A and 8B), MM 25.5.1078 is too fragmentary to assess skeletal articulation and completeness (S2 Table). Histological preservation is not optimal, with scarce dentine-specific response light under UV-A (e.g., only as shreds beneath the sockets in LT4 and LT3; Fig 9A). As for other RAV specimens preserved in sectioned and polished slabs, superficial erosion cannot be evaluated; however, numerous internal borings inside bones and teeth can be confidently identified as bioerosions (Fig 9B, 9E and 9F). These internal cavities can be elongated and tubular in shape, even contouring the dental curvature when affecting teeth (Fig 9F). Possible producers of such bioerosion might be boring sponges (e.g., *Entobia*) or endolithic marine fungi [91].

#### Description

MM 25.5.1078 is an approximately 40 X 20 cm polished slab of RAV (lower member) encasing sectioned jaws elements and associated teeth (Fig 8A). The slab thickness measures just 2.5 cm. Aside from a small unidentified bone fragment, the opposite side of the slab to the dentigerous elements bear no recognizable remains. A detailed description of the specimen is here provided for the first time:

*Cranium*. Two elongated dentigerous elements occupy the slab surface, perpendicularly positioned and sectioned one to another. The upper element is composed of 3 separate fragments, the larger one bearing four *in situ* teeth (Figs 8A, 8B, 9A–9C). Due to the limited exposed bone surface, we are unable to distinguish the anatomical provenance of this element. The lower isolated element is composed of two associated bones, only one being dentigerous (Fig 8A, 8B). The tooth-bearing bone is here interpreted as a sectioned portion of the right maxilla in dorsal view, with the anterior end facing the right fractured corner of the slab. The preserved maxilla bears five densely packed primary teeth, and laterally expands in the secondary palate floor. The end of the maxillary tooth row does not have broad maxillary shelves medially. The maxilla tapers posteriorly, and then remains incomplete. On the outer margin of the posterior end, a prominent neurovascular foramen with related fossa is visible throughout the longitudinal section, most likely representing an aperture of the maxillary division of the trigeminal nerve (Figs 8A and 9A). Posteriorly to the maxilla, an elongated arrow-shaped element articulates with the dentigerous ramus. The bone is here interpreted as the right jugal and appears not to overlap externally with the maxilla (Figs 8A and 9A); based on the concavities of the preserved maxilla, it perhaps was only overlapping the maxilla posterior (or superiorly) to the maxillary tooth row in the complete skull. Overall, we favor an interpretation of this complex as upper jaw because, if the teeth-bearing element was to be considered part of the dentary, the posterior element (splenial or angular) would have been more ventrally positioned rather than aligned.

*Dentition*. 15 teeth can be found on MM 25.5.1078. The numbering was arbitrarily started from the left corner of the slab with an X designation for loose teeth and a Y designation for teeth found inside sockets. Tooth buds or replacement teeth were labeled with an “a” after their corresponding tooth (Fig 8B). Teeth in the upper dentigerous ramus are longitudinally sectioned at different levels based on their alveolar position not being on a straight line (Fig 8A and 8C). The teeth TY1 and TY2 are the best representative for dental histological characterization in MM 25.5.1078, with clean cuts through the pulpal cavity (Fig 9C). TY1 shows a neighboring tooth bud (TY1a) in the early stage of development, still outside the primary tooth root (Fig 9C). TY5 to TY9 are all found in cross sections, with appreciable root-crown shapes and different stages of dental replacement (Figs 8A, 9A, 9E and 9F). The shape of the crowns in MM 25.5.1078 can be observed in apical view in TY8a and TY6a revealing that the crowns are moderately laterally compressed and with two distinct carinae nearly at a 180° one from another (Fig 9E and 9F). Beside TX1, teeth outside alveolar sockets are found in cross section.

#### Taxonomy

Taxonomic attribution of MM 25.5.1078 is challenging due to the limited preserved material: the specimen clearly shows a thecodont condition of the teeth, which is an archosaurian apomorphy [92]. This feature readily invalidates the historical attribution of MM 25.5.1078 to an ichthyosaur, because derived Ichthyopterygia present teeth with plicidentine set in continuous alveolar grooves (e.g., [93]); the absence of medio-posterior crypts for dental renewal can also readily exclude the attribution to Plesiosauria [94], leaving only marine crocodylomorphs as possible candidates among Jurassic marine reptiles. Tooth carinae are found in almost all members of Thalattosuchia, although some members of Teleosauroidea (such as *Machimosaurus*) have low or absent carinae [11, 10]. Alveoli are not densely packed as is typically observed in Teleosauroidea [10], and tooth size, despite being consistent with some Machimosaurini, appear closer to the metriorhynchoid condition. The depositional environment of the RAV, a mesopelagic setting opened to oceanic circulations, might exclude MM 25.5.1078 to be a coastal teleosauroid of large size (although we cannot preclude a specimen swept out in oceanic currents, or one that died during an oceanic migration). Moreover, more aquatic teleosauroid lineages (e.g., Aeolodontini) began to evolve in the Late Jurassic [12, 10]. Interestingly, the jugal in MM 25.5.1078 does not overlap the maxilla laterally (at least not in this particular section). In the metriorhynchoid clade *Zoneait* + Metriorhynchidae, the jugal clearly overlaps the posterior maxilla extensively, at least to the final two maxillary alveoli (e.g., *Zoneait nagorum* [14]; *‘Metriorhynchus’ brachyrhynchus* [2]; *Maledictosuchus riclaensis* [54]; *Torvoneustes coryphaeus* [95]; *Dakosaurus* [96]). In lateral view, this results in the posterior maxilla being largely obscured (other than the alveolar margin itself), but this is not the case in MM 25.5.1078. Moreover, the specimen exhibits large neurovascular fossae still adjacent to the end of the maxillary tooth row: the end of the maxillary tooth row is not preserved in the neotype of *Teleidosaurus calvadosii* [81] or in the holotype of *Eoneustes gaudryi* (NHMUK PV R 3353), but large neurovascular fossae on the maxillae are present ventral to the preserved jugals in both these species. Due to the paucity of recovered characters and because of the peculiar arrangement of the jugal in MM 25.5.1078, we decided to assign the specimen to Metriorhynchoidea *incertae sedis*, possibly outside the *Zoneait* + Metriorhynchidae clade.

### MGP-PD 27566: The teleosauroid of Ponte Serra

#### Systematic paleontology

Superorder: Crocodylomorpha Hay, 1930 [59]

Suborder: Thalattosuchia Fraas, 1901 [60] *sensu* [9]

Family: Teleosauridae Geoffroy Saint-Hilaire, 1831 [97]

Clade: Aeolodontini [98]

Locality and horizon: Ponte Serra (Belluno province, northeastern Italy: Fig 1), uppermost Kimmeridgian-lowermost Tithonian (Fig 2B), RAV upper unit (RAS) [34].

#### Age calibration and stratigraphic provenance

MGP-PD 27566 was assigned to the uppermost Kimmeridgian-lowermost Tithonian (Fig 2B) based on micropaleontological analyses and biostratigraphic classification of the source formation (see [34] for a detailed discussion on the calcareous nannofossil assemblage detected in the matrix of the specimen).

#### Taphonomy and preservation

MGP-PD 27566 is represented by axial, pelvic and dermoskeletal elements of a young teleosauroid attributable to the teleosaurid clade Aeolodontini found in a slab of upper member RAV datable at the uppermost Kimmeridgian-lowermost Tithonian [34]. Its description, anatomy and peculiar taphonomy (the specimen is preserved inside a regurgitalite bromalite) is already the focus of Serafini et al. [34], so we referred the readers to this study. Its preservational status (merging of skeletal elements, acid etching, gastric mucosa shielding from water dissolution) stands out from other thalattosuchians from the RAV deposits due to its unique taphonomic history [34].

#### Taxonomy

The taxonomic status of MGP-PD 27566 is here changed from Aeolodontinae (teleosaurid subfamily [10]) reported in Serafini et al. [34] to Aeolodontini (teleosaurid tribe) following the addendum of Johnson et al. [98].

### MGP-PD 32438: Unpublished specimen from Cima del Porco

#### Systematic paleontology

Superorder: Crocodylomorpha Hay, 1930 [59]

Suborder: Thalattosuchia Fraas, 1901 [60] *sensu* [9]

Superfamily: Metriorhynchoidea Fitzinger, 1843 [61] *sensu* [9]

Family: Metriorhynchidae Fitzinger, 1843 [61] *sensu* [9]

Metriorhynchidae indet.

Figs 10–12

**Fig 10:**
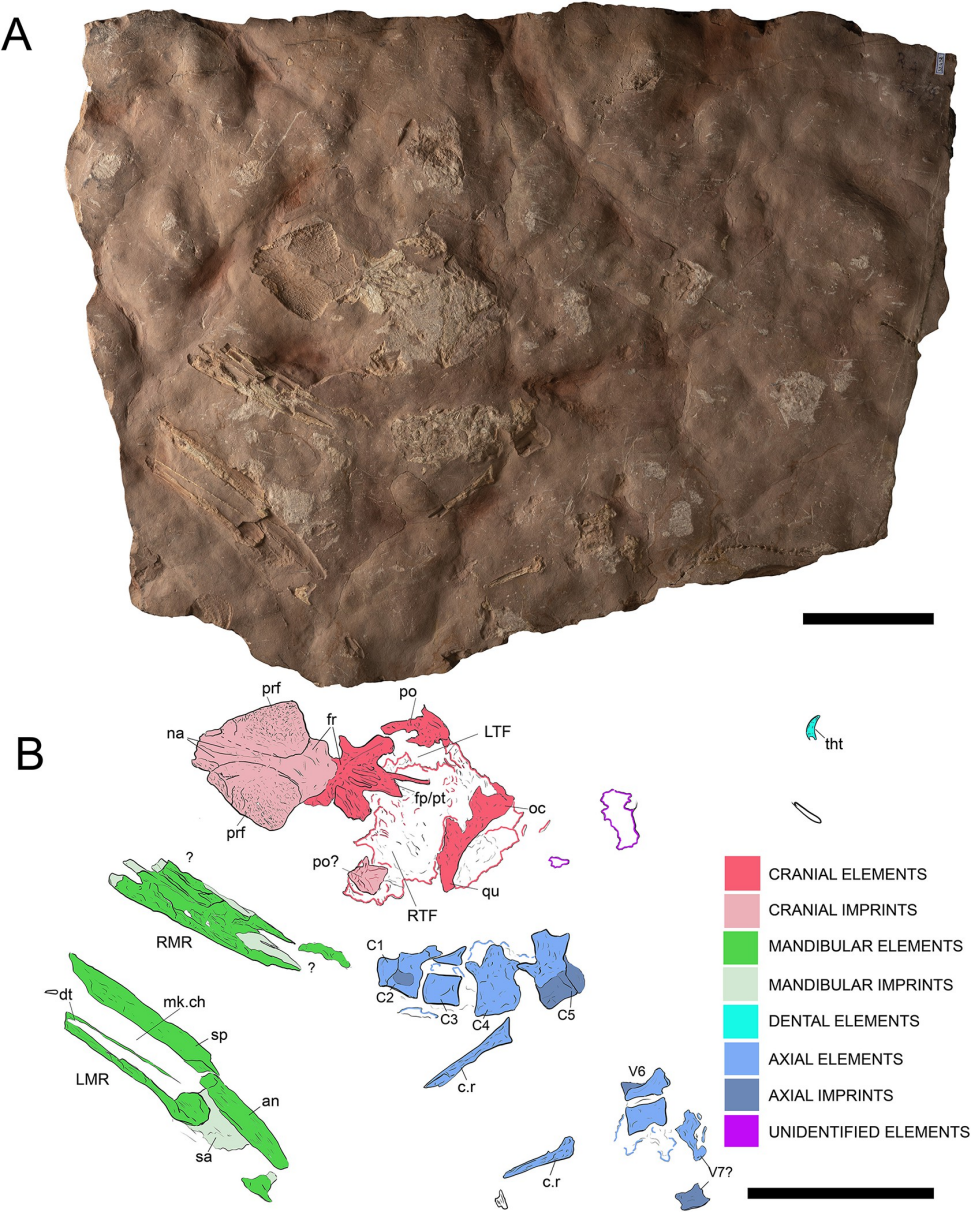
Overview of MGP-PD 32438. A) Orthogonal picture of the specimen. B) Anatomical drawing of the specimen with color-based differentiation of the main elements. Abbreviations: an, angular; C, cervical vertebra; c.r, cervical rib; dt, dentary; fr, frontal; fp/pt, frontoparietal-parietal; LMR, left mandibular ramus; LTF, left temporal fenestra; na, nasal; oc, occipital condyle; po, postorbital; prf, prefrontal; qu, quadrate; RMR, right mandibular ramus; RTF, right temporal fenestra; sa, surangular; sp, splenial; V, vertebra. Scale bars represent 10 cm.

**Fig 11:**
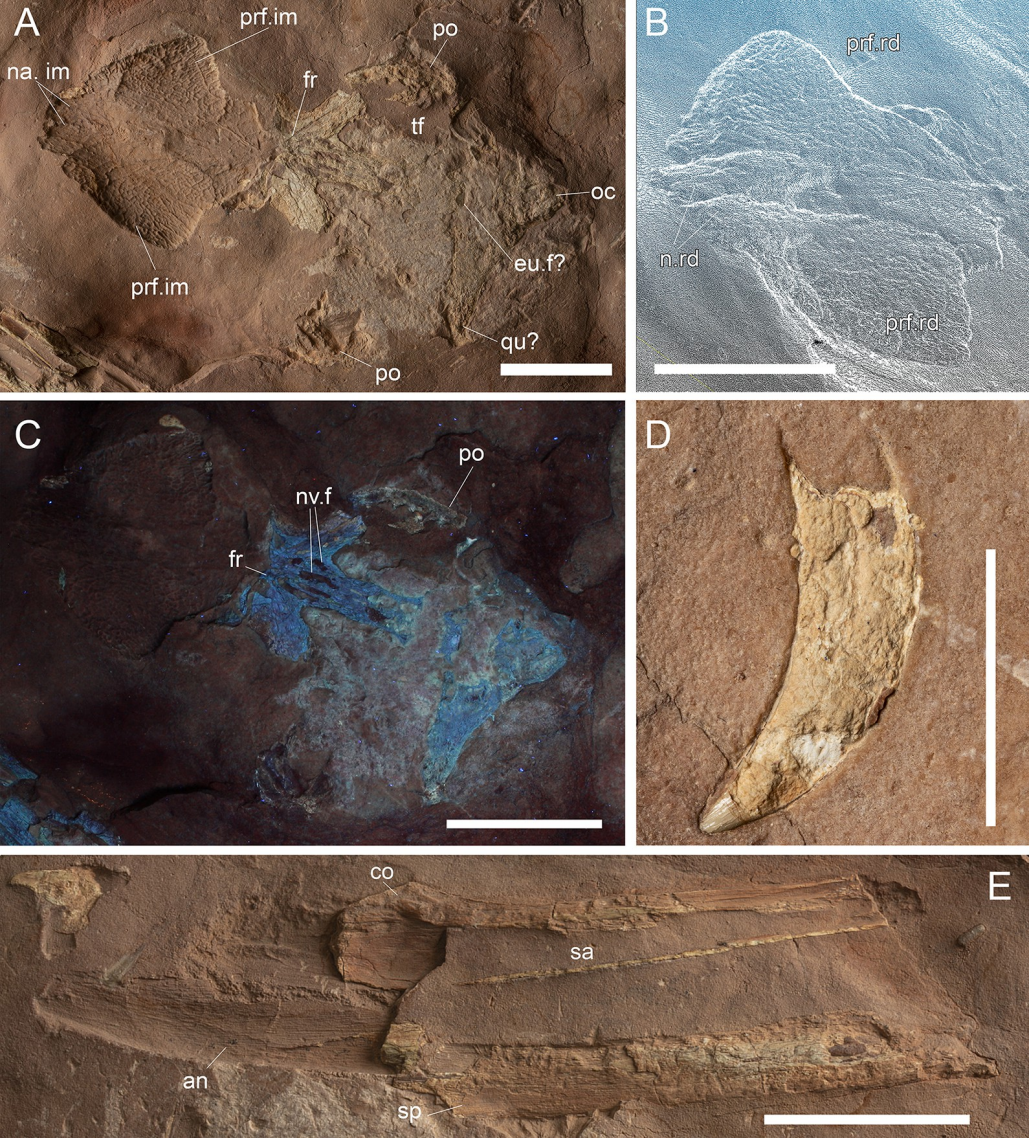
MGP-PD 32438 cranial details. A) Close-up of the skull roof and imprint. B) 3D rendition of the digitally flipped model of nasal/prefrontal imprints by means of photogrammetry. C) Skull roof elements under ultraviolet radiation (UVA-UVB-UVC). D) Close-up of the only tooth found with MGP-PD 32438. E) Close-up of the left mandibular ramus. Abbreviations: an, angular; eu.f, Eustachian foramen; fr, frontal; fr.f, frontal fossae; na.im, nasal imprint; n.rd, nasal 3D rendition; oc, occipital condyle; po, postorbital; prf.im, prefrontal imprint; prf.rd, prefrontal 3D rendition; sa, surangular; sp, splenial. Scale bars represent: A,B, E) 5 cm; D) 1 cm.

**Fig 12:**
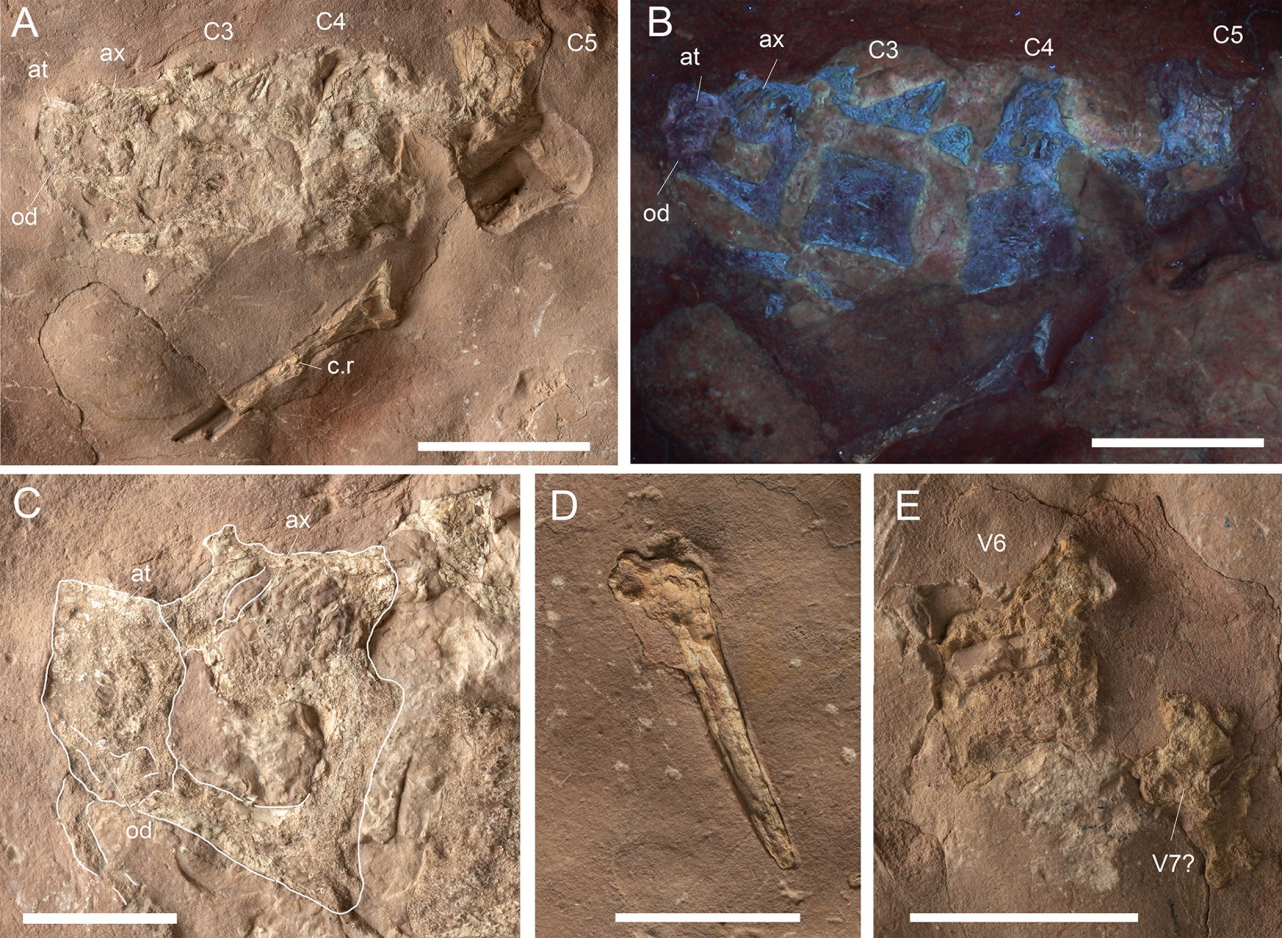
MGP-PD 32438 axial details. A) Close-up of the cervical vertebrae of the specimen. B) Cervical vertebrae under ultraviolet radiation (UVA-UVC). C) Close-up of the atlas-axis complex. D) Cervical rib close-up. E) Isolated sixth vertebra and possible remnants of a seventh. Abbreviations: at, atlas; ax, axis, C, cervical vertebra; c.r, cervical rib; od, odontoid process; V, vertebra. Scale bars represent: A,B,E) 5 cm; C) 2 cm; D) 3 cm.

Locality and horizon: Quarry at Cima del Porco (Asiago Plateau, Vicenza province, northern Italy; Fig 1), unknown horizon of RAV.

#### Age calibration and stratigraphic provenance

MGP-PD 32438 matrix was sampled for both petrographic and calcareous nannofossil analyses. The microfacies consists of a bioclastic packstone with abundant thin-shelled bivalves associated with micritic mud; among bioclasts, radiolarian and crinoidal remains are also present, along with iron oxides grains. The microfacies is consistent with that of the pseudonodular RAV lower unit (RAI), spanning from the upper Bajocian to the lower Callovian [26, 47]. A smear slide for calcareous nannofossil analysis was obtained from a brick-red marly layer still preserved on the lower side of the slab. MGP-PD 32438 displays a discretely diversified nannofossil assemblage consisting of *Watznaueria* aff. *W*. *manivitae* (R), *Watznaueria communis* (R), *W*. aff. *britannica* (F), *W*. *britannica* (R), *W*. *manivitae* (R), *W*. *barnesiae* (R), *Lotharingius sigillatus* (R), *L*. *hauffi* (R) and *Cyclagelosphaera margerelli* (R). The presence of *W*. *barnesiae* in the absence of *Cyclagelosphaera wiedmanni* allows the attribution of the sample to the NJT11 zone, which spans from the uppermost Bajocian to the upper Bathonian [90], consistently with the lithographic assignment to the RAI. MGP-PD 32438 is therefore assigned to the uppermost Bajocian-upper Bathonian (Fig 2A).

#### Taphonomy and preservation

MGP-PD 32438 is badly preserved, with extremely low values of cranial and axial completeness (S2 Table). The specimen landed dorsally (at least for the head) and subsequently experienced disarticulation of the lower jaws that drifted laterally (Fig 10A, 10B). Fluorescent lines dispersed on the slab under UV might imply the presence of other bones still embedded in the sediment, but for the time being we can only account for the presence of the specimen head and neck detached from the rest of the body, possibly by scavenging activities. From a histological perspective, MGP-PD 32438 skeletal tissues suffered both superficial erosion and lateral/dorsoventral compression, resulting in the majority of the compact bone to dissolve and trabecular structure of the cancellous component to collapse. As associated fossil fauna, rhyncholites are present near the remains, together with belemnite rostra.

#### Description

MGP-PD 32438 is represented by incomplete cranial and axial elements on an approximately 75 X 65 cm slab of finely grained RAV limestone (Fig 10A). The bones emerge from the upper polarity of the stratum. A detailed description of the specimen is here provided for the first time.

*Cranium*. Rostrally the skull is represented by detailed imprints in the matrix of the posterior midline portion of the nasals, dorsal surface of the prefrontals and anterior-most frontal (Figs 10B, 11A and 11C). Paired nasal imprints posteriorly reach the frontal, while laterally contacting the prefrontal imprints (Fig 11A and 11B). The prefrontals imprints are large and broad, posteromedially oriented and clearly tear-drop in shape (Fig 11A). A detailed network of coarse ornamentations is preserved in both impressions, progressively denser throughout the lateral margins. Flipping the 3D model of the nasal-prefrontal imprint, a digital rendition of the prefrontals becomes available, showing large and bulbous elements with complex surface texture (Fig 11B). The anteriormost portion of the frontal appears in both imprint and 3D model roughly arrowhead in shape, with distinctive lateral flanges bordering the posterior margins of the prefrontals. Posteriorly the actual frontal begins, almost entirely composed of the lateral and median processes, all three partially preserved (Fig 11A and 11C). Following the polarity of the stratum (and the concave imprints of the nasal-prefrontals), the frontal can be consequently considered to be in ventral view. Medial and lateral processes form an angle of about 50°, while the minimum width of the frontal between the orbits is 5 cm. The medial process of the frontal, at the beginning of the intertemporal (= fronto-parietal) bar, hosts four caudo-rostrally elongated pits (Fig 11A and 11C), the central two being the longest at 2.5 cm each in length. These fossae, distinctively rounded in their margins, appear to be arranged in a mirroring pattern, strongly suggesting an anatomical origin rather than a taphonomical artifact. Thalattosuchian frontals are rarely found isolated, and the ventral view is most often not visible, however, Hua [81] illustrated the ventral side of the frontal of *Teleidosaurus calvadosii*, which indeed presents two distinct fossae running in the central concavity of the bone ([81] Fig 2B). These fossae are likely indicative of vascular/neurovascular sites. Posteriorly to the frontal, multiple elements appear to be flattened in a single surface (Fig 11C); since the complex is ventrally placed, a contribution of the pterygoid might be present (merged during compression with upper elements). An arched bone is preserved on the left (*sensu* 180° flipped cranium) posterolateral margin: the bone could be interpreted as the left postorbital preserved as a fragmentary arch disconnected from the corresponding frontal lateral process (Figs 10B and 11A). On the opposite side, an impression and fragments of bone could belong to the right counterpart of this element. This arched element allows us to identify the outline of the almost complete left supratemporal fenestra (Figs 10B and 11A). Between fenestrae, bones are preserved as a merged, ventrodorsally compressed single surface, most likely as the remnant of the outermost compact tissue. A basisphenoid contribution could also be present. The occipital complex is preserved as a subtriangular isolated element, with the posterior apex should correspond to the occipital condyle (Figs 10A, 10B and 11A). In the reversed 3D model, a small notch can be observed anteriorly to the occipital condyle: judging from position itself, this notch could represent an imprint of the medial pharyngeal (Eustachian) foramen (Fig 11A). Medially to the occipital condyle, a small flange of the quadrate is preserved in ventral view (Figs 10B and 11A).

*Mandibles*. Two partial mandibular rami are found parallel to the skull axis, both partially embedded in the matrix (Fig 10A). The right ramus, closer to the other cranial elements, is too fragmented for a confident osteological interpretation (Fig 10B). The left ramus, though longitudinally sectioned, is more complete and is exposed by its medial side (Figs 10B and 11E). A section of the angular is posteriorly recognizable, weakly curved, possibly also with a small, subtriangular remnant of the retroarticular process. The surangular is badly preserved, while the coronoid process is more recognizable.

*Dentition*. The only tooth preserved on MGP-PD 32438 is a small, possibly anterior, isolated crown far from the skull elements (Figs 10B and 11D). The tooth, about 1 cm in length, shows a distinctive curvature apicobasally. Due to the strong erosion, it is difficult to assess the contact between crown and root, as only a few millimeters of the very tip still preserve distinguishable enamel. The tip shows light longitudinal striations that basally become more anastomosed (Fig 11D). No carinae are visible due to the crown being still embedded in the matrix.

*Vertebrae*. Six (potentially seven) vertebrae are found on the slab, five cervical still close to the anatomical articulation and a sixth centrum disassociated from the segment (Fig 10B; S1 Table). All centra are preserved in lateral view, associated with their relative neural arches and spines. The vertebrae appear slightly amphicoelic, with squared centra in lateral view; the direction of the preserved neural spines orient the segment with the anterior end close to the right mandibular ramus. C1 and C2 belong to the atlas-axis complex (Fig 12A–12C): the atlas CL is approximately half of the axis one, with the hypocentrum as the narrowest point; although not entirely visible in the lateral section, the odontoid process reaches the same size of the atlas hypocentrum (Fig 12C). The axis (C2) neural arch is square, with a small neural spine caudally projected. C3 to V6 share a very similar morphology, with short centra and prominent prezygapophyses (Fig 12A, 12B). C4 and C5 preserve the lateral shape of the neural arch, cranially projected in a prominent prezygapophysis, and of the neural spines, caudally projected and distally bifurcated; both features are typical of metriorhynchoid cervical centra [2, 99, 100]. C5 centrum is missing, represented only by a large imprint (Fig 12A). V6 is laterally sectioned, with a natural cast of the neural channel preserved below the arch (Fig 12E); the neural spine is short and squarish, consistent with the morphology of metriorhynchoid dorsal centra [99, 100]. A small vertebral element lies right below V6 centrum, possibly being its left lateral process. A squarish imprint at the border of the slab might represent a seventh vertebra (Figs 10B and 12E).

*Ribs*. Two disarticulated cervical ribs are preserved on the slab, both below the cervical segment (Figs 10A, 10B and 12D). In both elements the proximal end is preserved, but capitulum and tuberculum are not recognizable due to fragmentation.

#### Taxonomy

Despite the limited number of osteological elements with a strong preservation bias, inferences on the higher taxonomic status of MGP-PD 32438 can be made. The specimen is clearly a metriorhynchoid thalattosuchian and not an ichthyosaur as it was originally reported soon after its discovery [29], as the imprints of the prefrontals clearly represent broad, posteromedially oriented and teardrop-shaped bones that extensively participate in the orbit anterior margin [9, 64]. The prefrontal dorsal surface is greatly expanded laterally, overhanging part of the orbit (*Zoneait* + Metriorhynchidae clade apomorphy; [64]). MGP-PD 32438 exhibits characters unknown in early diverging metriorhynchoids more derived than *Magyarosuchus* (and the unnamed Toarcian taxon; [64]) such as a coronoid process considerably dorsal to the tooth-row (inferred even with the absent dentary; Fig 11E), the absence of external mandibular fenestrae (inferred from the disarticulated surangular where its ventral sutural margin is straight) and the absence of dorsal thoracic osteoderms (if considered non taphonomic in nature). An unambiguous apomorphy for Metriorhynchidae in MGP-PD 32438 is the presence of a true supraorbital notch [64]. If the odontoid is correctly identified, MGP-PD 32438 exhibits an intercentrum subequal in length to atlas centrum, another apomorphy of Metriorhynchinae *sensu* [9] (or the *Thalattosuchus* + Metriorhynchinae clade; MTY preliminary observations). The specimen can be therefore confidently assigned to Metriorhynchidae. Additionally, the narrow angle between medial and lateral processes of the frontal is acute (50°), closer to the condition of *Cricosaurus* (45°; [4, 9]); this feature might be indicative of the specimen being a metriorhynchine. It is noteworthy that MGP-PD 32438 also has features that are previously unseen in other metriorhynchids: the prefrontals appears tightly close, spaced by less than 2 cm of nasals at the apex of the lateral inner margin; such reduced space between prefrontals is not found in any other metriorhynchids (e.g., [2, 4, 9, 20, 54, 101]) especially not in the coeval and sympatric *Neptunidraco* [25], possibly representing an autapomorphy of MGP-PD 32438 itself. The taxon that shares most similarities in the prefrontals shape and spacing with MGP-PD 32438 is currently *Maledictosuchus riclaensis*, a metriorhynchine from the Callovian of Spain [54], that also preserves axial elements generally similar to the ones in the studied specimen (see [102]). Unfortunately, MGP-PD 32438 presents too limited and fragmentary osteological features for considering the erection of a new taxon, or to reliably score for phylogenetic analysis (i.e., is the absence of osteoderms a true absence or taphonomic bias?). Nonetheless, due to its Bajocian-Bathonian age, the specimen from Cima del Porco is one of the oldest known representatives of Metriorhynchidae, alongside with *N*. *ammoniticus*.

### FOS03839: The Rovereto specimen

#### Systematic paleontology

Superorder: Crocodylomorpha Hay, 1930 [59]

Suborder: Thalattosuchia Fraas, 1901 [60] *sensu* [9]

Superfamily: Metriorhynchoidea Fitzinger, 1843 [61] *sensu* [9]

Metriorhynchoidea indet.

Fig 13

**Fig 13:**
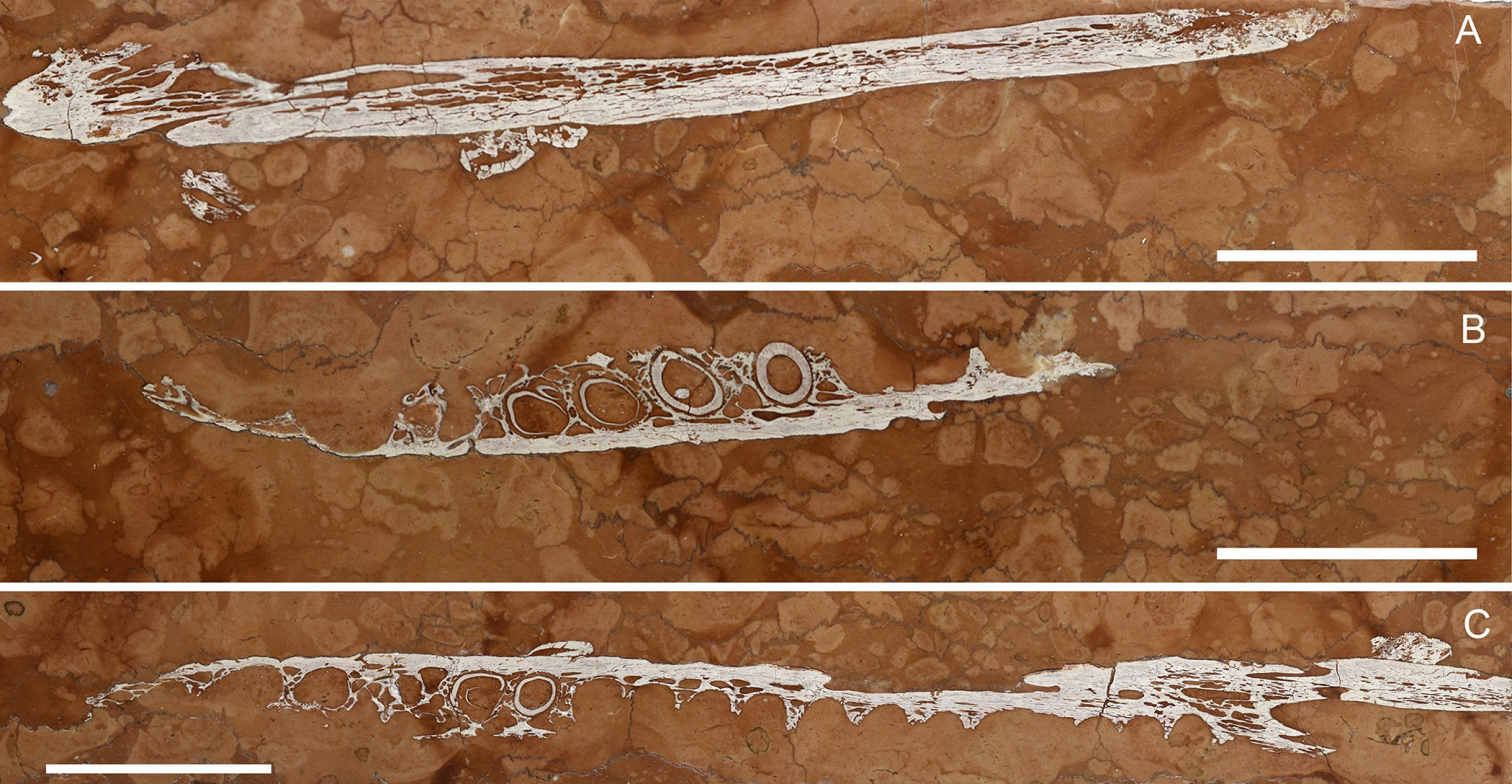
Overview of FOS03839. A) Sectioned mandibular(?) ramus. B-C) sections of the same mandibular ramus according to Cau (2019). Scale bars represent 5 cm.

Locality and horizon: Sasso d’Asiago (Asiago Plateau, Vicenza province, northern Italy; Fig 1), Bajocian-Callovian lower unit of RAV (RAI), according to Bizzarini [28] and Mongiovì et al. [41].

Age calibration and stratigraphic provenance:

The lower unit provenance was confirmed by petrographic sections which revealed the typical RAI microfacies attributable to the upper Bajocian-lower Callovian interval (Fig 2A: see paragraphs above; [47]).

#### Taphonomy and preservation

FOS03839 is described by Bizzarini [28] and Cau [31, 52] as mandibular rami (Fig 13A–13C) on six sectioned slabs of lower member RAV (Bajocian-Callovian). FOS03839 scores very low in skeletal completeness, overall being more recognizable as isolated fragments. Elements continuity between slabs is difficult to assess, as the cuts are too distant from one another. Superficial erosion cannot be evaluated but the sectioned skeletal tissue is internally better preserved than most other RAV crocodylomorph specimens from the lower RAV units; teeth and bone do not show sign of internal bioerosions (Fig 13B) and nutrient foramina can still be found undeformed and clear of matrix infillment (Fig 13C).

#### Taxonomy

The specimen was preliminary assigned to Geosaurinae and to *Neptunidraco* sp. by Cau [52] based on alveolar and dental features (17–19 dentary teeth, narrow interalveolar spacing, anterior alveoli larger than posterior ones; [52]). However, later Cau [31] found these characters to be widespread among Metriorhynchoidea and therefore not reliable for a genus-level attribution; the specimen was consequently assigned to Metriorhynchoidea *incertae sedis*. We refrain from adding changes to the anatomical and taxonomical discussion of the specimen as FOS03839 is currently being independently revised.

### MCLSC T2: Unpublished Chioggia specimen

#### Systematic paleontology

Superorder: Crocodylomorpha Hay, 1930 [59]

Suborder: Thalattosuchia Fraas, 1901 [60] *sensu* [9]

Superfamily: Metriorhynchoidea Fitzinger, 1843 [61] *sensu* [9]

Metriorhynchoidea indet.

Figs 14–16

**Fig 14:**
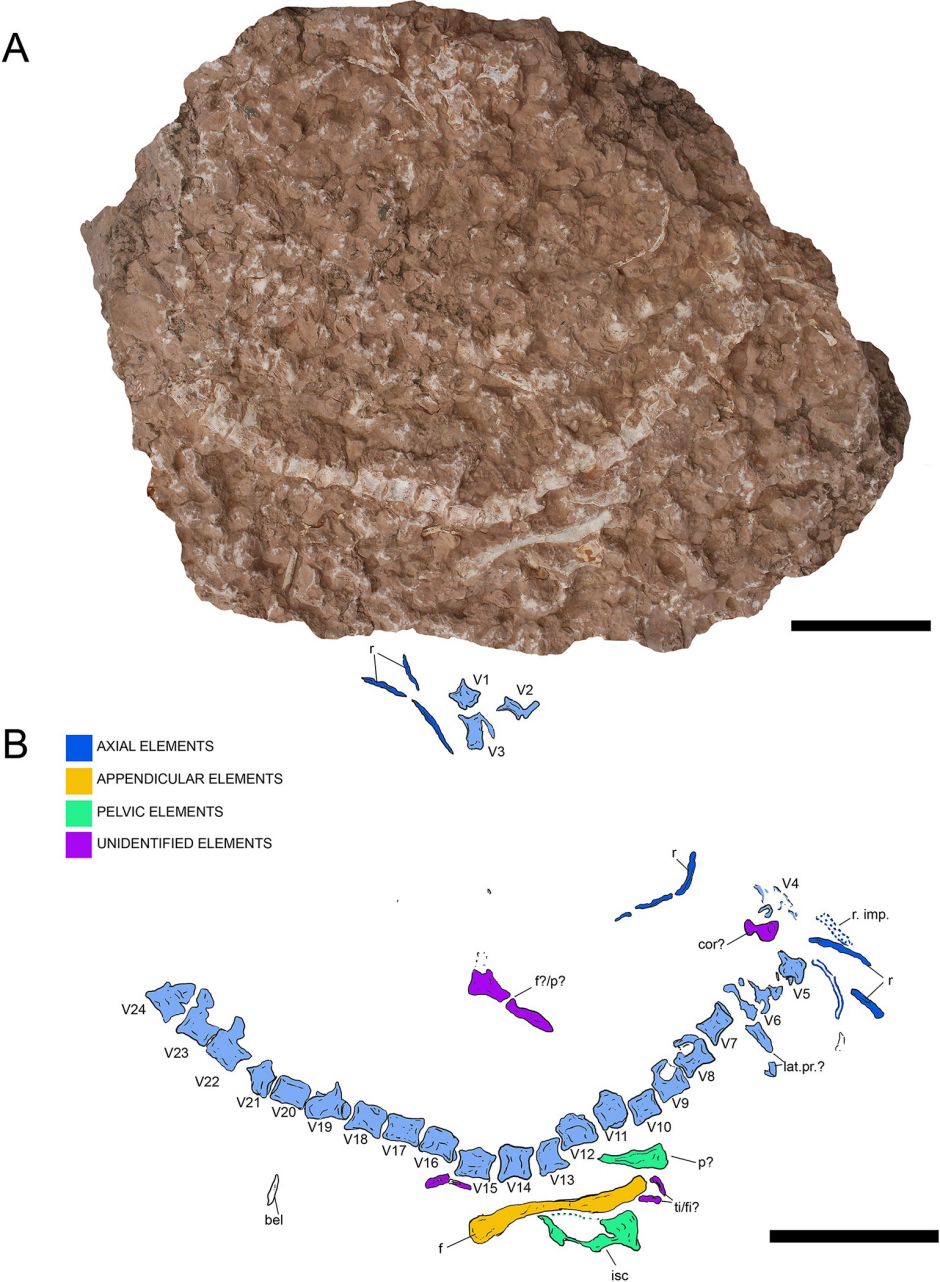
Overview of MCLSC T2. A) Orthogonal picture of the specimen. B) Anatomical drawing with color-based differentiation of the elements. Abbreviations: cor, coracoid; f, femur; fi, fibula; lat.pr, lateral process; isc, ischium; p, pubis; t, tibia; r, rib; r.imp, rib imprint; V, vertebra. Scale bar represents 20 cm.

**Fig 15:**
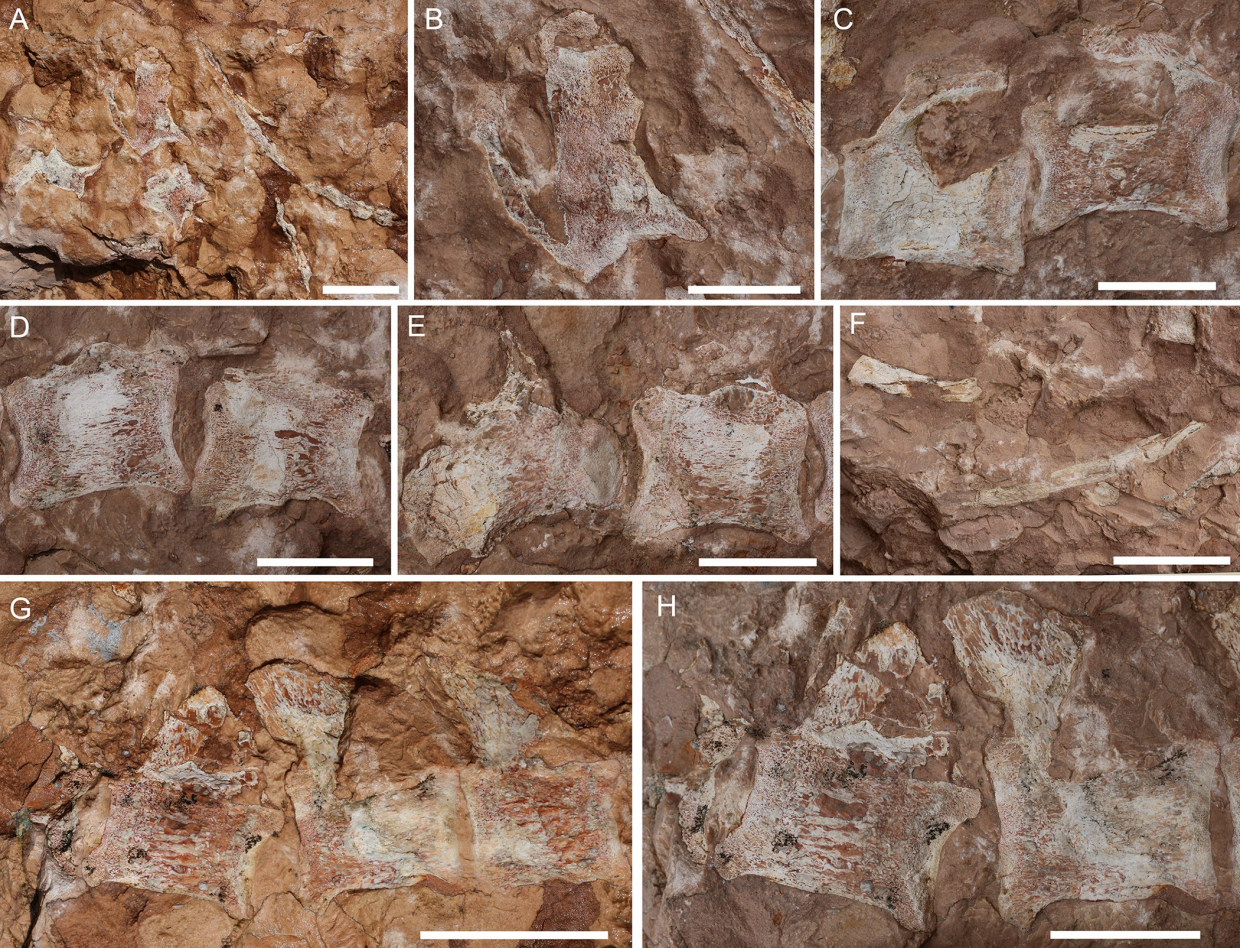
MCLSC T2 axial detail. A) Isolated anterior vertebrae and ribs. B) Close-up of V3. C) Close-up of two anterior centra (from the left) V9 and V8. D) Close up of two middle vertebrae (from the left) V17 and V16. E) Close-up of two posterior centra (from the left) V19 and V18. F) Isolated anterior ribs and rib imprint. G) Last three preserved posterior centra of MCLSC T2. H) Close-up of the last two centra V23 and V22 (from the left). Scale bars represent: A, F, G) 5 cm; B, C, D, E, H) 3 cm.

**Fig 16:**
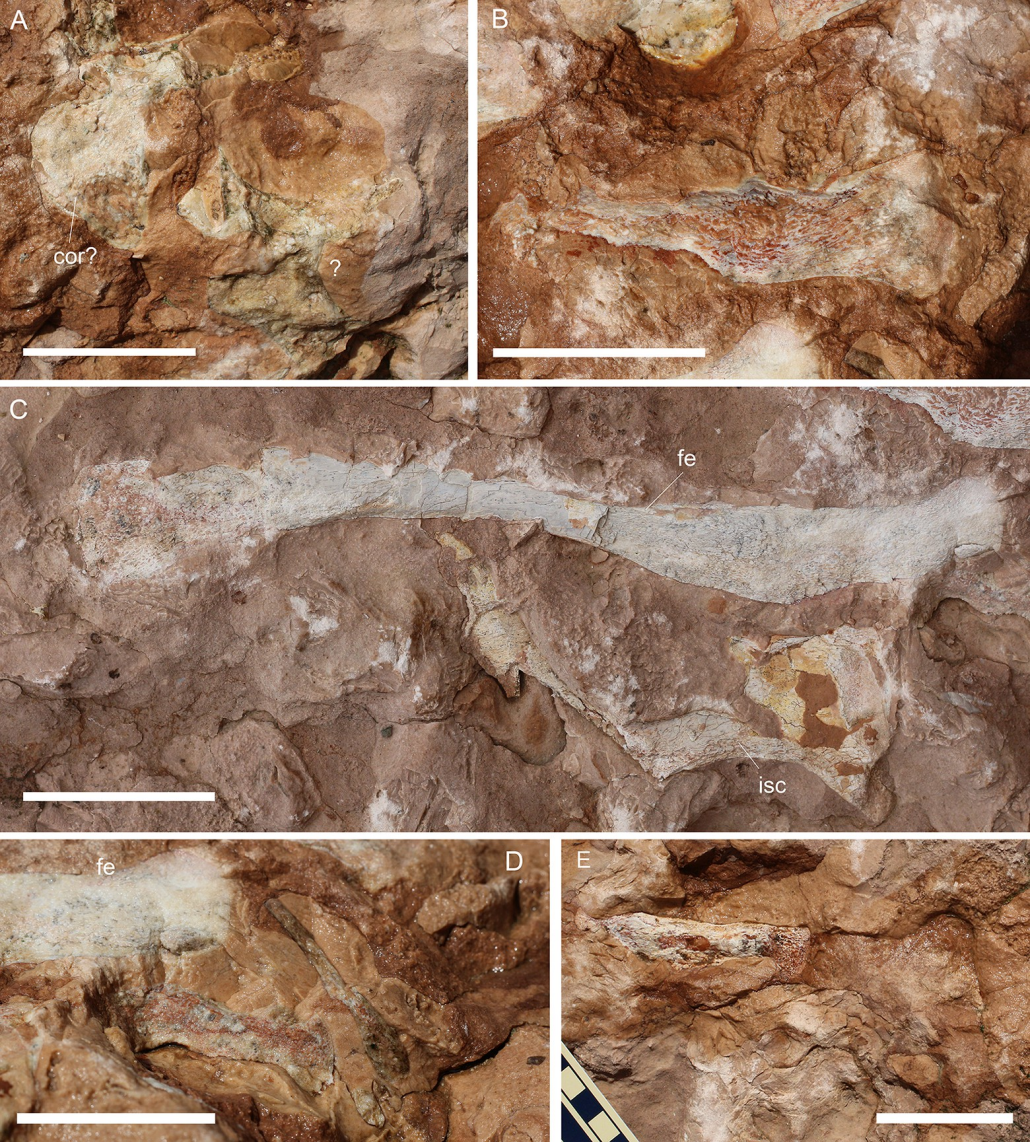
MCLSC T2 appendicular details. A) Putative coracoid and unidentified flat elements. B) Preserved pubis close-up. C) Femur and ischium close-up. D) Partially outcropping long elements close to the femur, possibly representing tibia and fibula. E) Unidentified isolated bone and imprint, possibly representing the other femur or pubis. Abbreviations: cor, coracoid; fe, femur; isc, ischium. Scale bars represent: A) 4 cm; B-E) 5 cm.

Locality and horizon: Possibly Sasso d’Asiago (Asiago Plateau, Vicenza province, northern Italy: Fig 1). Age and horizon of provenance originally unknown. Herein considered to be middle-late Bajocian in age.

#### Age calibration and stratigraphic provenance

The thin section obtained from MCLSC T2 matrix shows a bioclastic packstone dominated by densely packed thin-shelled bivalves associated with micritic mud, radiolarians and remains of echinoderms. Such microfacies is compatible with that of the pseudonodular facies of the RAI (upper Bajocian-lower Callovian; [47]). Calcareous nannofossil data obtained from a thin marly layer still preserved on the upper side of the boulder allowed to further constrain the interval of provenance of the finding. The assemblage is scarce and badly preserved; however, the following taxa were recognized: *Watznaueria* aff. *W*. *manivitae* (F), *W*. *gaetanii* (F), *W*. *britannica* (R), *W*. *communis* (R) and *W*. *manivitae* (R). Because of the presence of *W*. *manivitae* in the absence of *Carinolithus superbus* and *Watznaueria barnesiae*, the assemblage can be ascribed to the calcareous nannofossil subzone NJT10b [89], therefore referable to the middle-upper portion of the Bajocian (Fig 2A). Lithology and biostratigraphic dating allow us to firmly assign the specimen to the lower portion of RAI.

#### Taphonomy and preservation

Most elements in MCLSC T2 are found partially articulated (Fig 14A) or very close to the anatomical position (i.e., vertebral succession of 20 centra and pelvic girdle alongside the hindlimb elements: Fig 14B; S2 Table). Only the posterior end of the animal is preserved, with only disarticulated and disassociated anterior elements. The articulated portion of the vertebral column is dorsally curved (about 125°); an opisthotonic arrangement of axial elements is therefore deductible from the remains of the specimen. The taphonomic explanation of the opisthotonic position (or death pose) is controversial, either seen as a sign of a damage in the central nervous system of the animal (e.g., cerebral infections) resulting in the spastic perimortem contraction of the column muscles [103] or simply justified by postmortem ligaments modification due to maceration and/or desiccation [104]. The phenomenon is poorly documented in deep-water setting, and MCLSC T2 does not offer evidence for either major theories; the upward bending of the vertebral column of the specimen could also be just the result of the carcass arching during drifting or floating [105]. The bone tissue is heavily eroded to the cancellous component, with evident infillment of matrix and trabecular collapse (Fig 15). Tens of nautiloid rhyncholites can be found on the surface of the boulder (23 mapped only close to the bones), many of them very close to the skeletal elements. Other associated invertebrates are represented by ammonite aptychi and belemnite elements, although their limited presence could be coincidental. Peculiar circular borings infilled by gray calcite can be found in some sectioned centra (Fig 15B, 15C and 15G).

#### Description

MCLSC T2 is represented by axial, pectoral, pelvic and appendicular elements of a metriorhynchoid outcropping from a large RAV boulder (Fig 14A and 14B). Despite the boulder being inside the Chioggia’ civic museum cloister since the 1990’s, its importance was only noted in 2022 by GS during a collection survey. The specimen was found in a seaway dam in Pellestrina (Lagoon of Venezia) most probably in the late 1990’s. While there is no official account for MCLSC T2 discovery and its moving, another marine tetrapod (an ichthyosaur; [29]) was found in the same way (and same place) and moved to the Chioggia Museum in 1997. MCLSC T2 was most likely found and moved together with the ichthyosaur. Here we present its first official description.

*Vertebrae*. 24 vertebrae can be identified on the surface of the boulder, 3 isolated centra and 20 still articulated (Fig 14B). The articulated segment of the column is preserved in lateral view, with almost all elements laterally sectioned by erosion. Thanks to the last three articulated vertebrae with preserved partial neural spines (Figs 14A, 15G and 15H), both the antero-posterior and dorsoventral orientation of the specimen can be recognized. No complete lateral processes nor haemal facets can be recognized in any of MCLSC T2 centra. Numbering arbitrarily started from the isolated centra and then caudally continued in the articulated column. We avoided the distinction of regionalized portions of the preserved column due to the insufficient state of preservation of the elements (for the majority still embedded in the matrix). For this reason, vertebrae are labelled as just “V”. V1 appears squarish, short and laterally oriented, while V2 is seemingly anterolaterally oriented, with a preserved fragment of the neural spine (Figs 14B, 15A, 15B). The articulated elements are instead preserved in lateral view (Fig 15C, 15D, 15G and 15H), with the only exception of V19 which features a visible anterior condyle (Fig 15E), appearing slightly more anteriorly tilted than the rest of the segment. Overall centra throughout MCLSC T2 are rectangular in profile, longer than taller and slightly amphicoelic. Some elements can be found with portions of neural arches still attached to the centra (e.g., V4-5-7-17) but V22-23–24 are the only vertebrae with most of the neural arch and neural spine outlines still preserved (Fig 15G and 15H). Neural spines from these three vertebrae are dorsally narrow and the neural arches do not show prominent pre-zygapophyses. V22 and V23 share the same caudal orientation of the neural spines, while the one from V24 points cranially, in the opposite direction (Fig 15G). Rather than representing the flexural region (area of the tailbend in metriorhynchid characterized by opposite-oriented neural spines; e.g., [1, 21, 22]), this sudden change in neural spines orientation is explained by a partial preservation of V24 neural spine (Fig 15H). Unfortunately, a confident identification of the preserved column regions is made unreliable by preservation. In the same perspective, despite a bias on the measures due to a relevant overlay of matrix on the elements, it can be observed a descendant trend toward the anterior vertebrae in CL values (S1 Table); the last three vertebrae, seem to increase both CL values (48,3<52<53) and CH values (34<35,1<36,6).

*Ribs*. Five ribs are preserved on the surface of the boulder (Fig 14B). Two appear to laterally outcrop next to V1 and V3 (Fig 15A), while the remaining three can be found at the beginning of the articulated column (Fig 15F). No capitula/tubercula are preserved; in a possible rib imprint a central groove can be observed running longitudinally to the costal shaft. A large long bone can be found ventrally to the column that could be identified as a rib (Figs 14A and 15B), however, due to preservation, an appendicular interpretation should also not be easily ruled out.

*Girdles and appendicular elements*. Cranially to the articular portion of vertebral column a small and rounded element (Figs 14B and 16A) can be recognized among other unidentified structures still embedded in the matrix (most likely vertebral fragments): the rounded object might be recognized as a partial coracoid displaced from the scapular girdle, but this interpretation is dubious. Ventrally to the mid-column, three elongated and flat elements stand out from the centra and the nodular texture of the matrix (Figs 14A and 14B, 16B and 16C): from below these are recognized as a partial ischium (Fig 16C) still deeply embedded in the sediment, a largely complete right femur (Fig 16C) and possibly a fractured pubis (Fig 16B). The ischium is trapezoidal, with a reduced anterior process lacking clear articulation facets. The femur is slightly sigmoidal in profile and with a distinctive medio-distal crest not far from the articular facet. Both preserved epiphyses are small and rounded (although fractured and partially embedded), while the mid diaphysis appears remarkably thin. Near the femur distal epiphysis, two elongated bones of small sizes can be seen closely associated (Fig 16D); it is unclear whether the corresponding tibia and fibula could still be embedded in the matrix. Dorsally to the column, a last long bone element outcrops from the matrix, possibly a portion of the other femur or pubis (Fig 16E).

#### Taxonomy

The taxonomic status of MCLSC T2 is difficult to evaluate; major taxonomic characters in Metriorhynchoidea are based on cranial morphology, while axial and appendicular elements are less diagnostic–due to the paucity of early diverging metriorhynchoids with reasonably complete postcrania (e.g., [4, 14–16, 64, 81, 106, 107]). Moreover, skeletal elements from MCLSC T2 are far from complete, most often still embedded in the surrounding matrix hampering a confident morphological characterization. The only diagnostic characters that can be scored from the specimen are the following: the absence of dorsal caudal osteoderms (apomorphies unknown in early diverging metriorhynchoids more derived than *Pelagosaurus*; [64]); ischium anterior processes reduced in size and lacking clear distinct articulation facets for both the ilium and the pubis, femur distal condyles incompletely ossified (but equivocal due to the poor preservation), absence of dorsal thoracic and gastral osteoderms (characters unknown in early diverging metriorhynchoids more derived than *Magyarosuchus* and the unnamed Toarcian taxon; [64]). The only known thalattosuchians that share all these diagnostic characters are members of Metriorhynchidae (although, the presence/morphology of these characters cannot be assessed, so excluded, for *Eoneustes* and *Zoneait*). The absence of osteoderms could be taphonomical, however, the semiarticulated nature of the specimen’s column, together with the preservation of dissociated ribs seems indicative of little disturbance of the carcass at the seafloor; therefore, at least some dermoskeletal elements would have feasibly been preserved on the boulder surface, if originally present. The femur is 24 cm long, smaller than that of the early diverging metriorhynchoid *Magyarosuchus fitosi* (35 cm; [15]) and the derived geosaurine *Torvoneustes carpenteri* (34 cm; [108]) but longer than some derived metriorhynchines (e.g., *Cricosaurus albersdoerferi*, 15–16 cm; [22]). Its peculiar sigmoidal profile and blade-like epiphyses have never been described in other metriorhynchoids: it could either be a taphonomical artifact (partial erosion, matrix encrustation) or it might well represent its own autapomorphy. Sectioned vertebrae in MCLSC T2 are highly osteoporotic, an inner microstructure indicative of a strong pelagic adaptation [109]; a derived pelagic lifestyle is well known in Metriorhynchidae. However, during the land-to-sea transition within Metriorhynchoidea there was a mosaic acquisition of these characters, with taxa becoming progressively more aquatic (e.g., [14, 15, 17]). Bajocian metriorhynchoid genera (*Teleidosaurus*, *Eoneustes*, *Zoneait*) have a scarce and dispersed fossil record [81] and little is known about their axial and hindlimb morphology [4, 14, 81]. With the available characters we assign MCLSC T2 to Metriorhynchoidea indet., although we cannot preclude the possibility that once the timing of these postcranial adaptations is better understood the specimen could be re-interpreted as the oldest known metriorhynchid.

Results of our study on the known and available thalattosuchian record from the RAV are summarized in the table below (Table 1).

## Discussion

### Biostratigraphic trends of the RAV thalattosuchian record

Of the eight documented thalattosuchian specimens from the RAV, five are recovered from Middle Jurassic units, and three (considering the Cesuna vertebrae as possible thalattosuchian) are from the Upper Jurassic (Fig 2). For the first time we consistently applied calcareous nannofossil analysis to sort the marine crocodylomorph record from the RAV. Our data suggest that the calcareous nannofossils are a key biostratigraphic tool in the RAV setting, especially when paired with petrographic characterization of the source matrix lithofacies [58, 47]. Moreover, the added benefit of this approach is the limited amount of loose material needed for extraction, pivotal when dealing with such rare specimens. All three newly described specimens (i.e., MM 25.5.1078, MGP-PD 32438, MCLSC T2) were successfully assigned to a restricted time interval between the middle Bajocian and the upper Bathonian. To this date the only other marine reptile retrieved from the lowermost unit of the RAV Fm. (Bajocian-Bathonian) is a pliosaurid plesiosaur ([32]; G.S. & L.G. preliminary observation). Marine tetrapods of continental Europe are generally rare in this time interval, and the presence of predominantly marine crocodylomorphs in the Middle Jurassic of the RAV is noteworthy. However, any major discussion on the possible ecological prevalence of thalattosuchians between marine reptiles in the Middle Jurassic of this portion of the Western Tethys is biased by the extremely limited record of vertebrates in the RAV.

#### Preservational bias

All thalattosuchian specimens from the RAV are in a bad preservation state typical of the taphonomic regime of the deposit. The slow sedimentation rates of RAV [47] ensured a prolonged exposure of the carcasses on the seafloor and the well oxygenated bottom conditions allowed the establishment of scavenging and opportunistic communities. However, we refrain from discussing deadfall ecology here as it is currently the subject of a larger survey on all marine tetrapods from the RAV (Serafini et al., preliminary observations). Other marine reptiles from the RAV share the same taphonomic pattern [33], but the crocodylomorph record is particularly indicative. In addition to scavenging and carcass exploitation, water dissolution appears to have greatly affected each histological component of the specimens’ skeletal tissues: the compact bone is rarely found intact, most often strongly eroded down to the cancellous component. In the same way the enamel, despite its structural integrity, is often found absent in teeth in contact with the water-sediment and is instead found preserved only in replacement crowns still embedded in bone or in to-be-replaced teeth (e.g., the Chiampo specimen, MM 25.5.1078). In addition, some specimens also show heavy signs of lithostatic compression (e.g., MGP-PD 32438), with the merging of skeletal elements and the collapse of the trabeculae in the spongy bone. Disarticulation is less predominant in the RAV specimens, with many specimens found with still valuable degree of connection between elements (S2 Table). This condition is possibly indicative of seafloor deposition less disturbed by currents, as previously speculated [26, 47].

With the contribution of all these postmortem modifications, all thalattosuchian specimens from the RAV are too taphonomically biased for major taxonomic assessment. We recognize the taxonomic potential of each specimen, with some of them being probably genera and species different from their coeval or subsequent European relatives, (especially the Bajocian specimens) but we cannot confidently score their characteristic in a reliable diagnosis. This preservational bias has major consequences for future evolutionary and biogeographical studies on this fossil record.

#### Paleobiogeographic implication

Without adequate taxonomic identifications, most of the RAV thalattosuchian record fails to add major insights on the evolutionary timing of Metriorhynchoidea in the Middle-Upper Jurassic. However, the RAV record enriches our knowledge on the biogeography of the group during the undersampled Bajocian-Bathonian interval of the Tethys Ocean. MM 25.5.1078 and MCLSC T2 testify to the presence of metriorhynchoids in the western side of the Tethys since the upper Bajocian; the only other metriorhynchoid taxon from this stage in Europe (France) is *Eoneustes gaudryi* [4, 80, 81], while *Zoneait nargorum* might range to a slightly older horizon in North America (latest Aalenian-lower Bajocian; [14]). It is interesting to note that while both *Eoneustes* and *Zoneait* (together with the later *Teleidosaurus* from the Bathonian of France) are found in relatively shallow-water deposits [14, 80, 81], the RAV specimens are found in a mesopelagic setting open to oceanic circulation [47]. This might confirm the colonization of open-sea environments by metriorhynchoids already in the Middle Jurassic. The osteoporotic-like texture of the cancellous bone in MCLSC T2 would seemingly support this scenario, with histological evidence directly linked to strong aquatic adaptations. MGP-PD 32438 and *Neptunidraco ammoniticus* are the oldest demonstrably metriorhynchid specimens known, both coming either from uppermost Bajocian or lowermost Bathonian. More interestingly, they are seemingly members of the two primary subdivisions of the group (Metriorhynchinae and Geosaurinae *sensu* [9]). Therefore, the origin of Metriorhynchidae must be pre-Bathonian. Moreover, our study supports the hypothesis that the taxonomic diversity observed in the Callovian metriorhynchids of western Europe (with members of Metriorhynchinae and Geosaurinae being present, e.g., [2, 4, 95, 99]) was likely the result of the marine transgression at the beginning of the Callovian expanding the geographical range of pre-existing clades, and not the origination of the clades themselves.

Frustratingly, we are unable to determine whether the Bajocian specimen MCLSC T2 was a metriorhynchid or a closely related early diverging metriorhynchoid. It displays a suite of postcranial characters currently only known amongst metriorhynchids, but the paucity of postcranial metriorhynchoid remains from the lower Middle Jurassic (Aalenian-Bathonian; [14, 16, 80, 81, 110, 111]) means that the evolutionary timing of these features remains obscured. However, we can conclusively say that a metriorhynchid-like postcranial skeleton has been found from a mesopelagic depositional environment during the Bajocian, and regardless of whether this taxon was a metriorhynchid (*sensu stricto*), it suggests that metriorhynchoids had made the transition to being fully aquatic by the middle-upper Bajocian.

The Upper Jurassic portion of the thalattosuchian RAV record is much more in line with the rest of Europe, with both Oxfordian metriorhynchid (MGP-PD 26552) and Kimmeridgian-Tithonian aeolodontin teleosauroid (MGP-PD 27566), common faunal component in epicontinental seas and deep-water settings of Europe in their respective stages [24, 10, 99].

## Conclusions

Here we present the first biostratigraphic sorting and taphonomic assessment of the RAV fossil record of Thalattosuchia, together with the description of three new specimens and revision of older material. MM 25.5.1078 is here identified as bioeroded dentigerous elements of a metriorhynchoid from the upper Bajocian of Asiago. MGP-PD 32438 is here identified as a metriorhynchid from the upper Bajocian-upper Bathonian of Cima del Porco (Asiago Plateau); despite its poor preservation, the specimen shows a remarkable detail of the cranial anatomy in ventral view. Together with *Neptunidraco ammoniticus*, MGP-PD 32438 is the oldest representative of Metriorhynchidae known to date. MCLSC T2 is here described as the axial skeleton and pelvic/hindlimb elements of an undetermined metriorhynchoid from the upper Bajocian of (most likely) Sasso d’Asiago. The shape of the femur of MCLSC T2 shows features previously unseen in Metriorhynchoidea, and a future preparation could possibly allow its scoring as an autapomorphy. The specimen is also peculiarly arched dorsoventrally in an opisthotonic condition not common in deep-water taphonomy. We also provide a revision of the historically relevant “*Steneosaurus*” *barettoni*, identifying the specimen as a mid-to-upper Oxfordian metriorhynchid with close resemblance to Geosaurinae. From our taphonomic analysis we found the thalattosuchian record of the RAV to be too preservationally biased for any reliable taxonomic assignment below the family level. Regardless of such uncertainties, the RAV record offers major biogeographic insights for the open-ocean transition of Metriorhynchoidea during the Middle Jurassic.

## Supporting information

S1 Table Axial measurements for MGP-PD 32438 and MCLSC T2. Abbreviations: CL, centrum length; CH, centrum height.
(XLSX)

S2 Table Scoring for taphonomic values on the dataset. Scoring is from 0 to 4 (0% = 0; 1–25% = 1; 25–50% = 2; 50–75% = 3; 75–100% = 4); C = completeness (scoring for percentage of completeness for each anatomical unit); A = articulation (scoring for percentage of articulation for each anatomical unit); E = erosion (scoring for percentage of eroded compact bone for each anatomical unit).
(DOCX)
