# Supplementary material for: Reappraisal of the thalattosuchian crocodylomorph record from the Middle-Upper Jurassic Rosso Ammonitico Veronese of northeastern Italy: Age calibration, new specimens and taphonomic biases
Source: PLoS One. 2023 Oct 30;18(10):e0293614. doi: 10.1371/journal.pone.0293614 (PMC10615311; doi:10.1371/journal.pone.0293614)
Supplement: S2 Table — Scoring is from 0 to 4 (0% = 0; 1–25% = 1; 25–50% = 2; 50–75% = 3; 75–100% = 4); C = completeness (scoring for percentage of completeness for each anatomical unit); A = articulation (scoring for percentage of articulation for each anatomical unit); E = erosion (scoring for percentage of eroded compact bone for each anatomical unit). (DOCX) [file pone.0293614.s002.docx]

|  |  |  | **Anatomical units** |  |  |
| --- | --- | --- | --- | --- | --- |
|  |  |  |  |  |  |
| **Specimen** | **Skull** | **Anterior column** | **Posterior column** | **Forelimbs/girdle** | **Hindlimbs/girdle** |
|  |  |  |  |  |  |
| MGP-PD 26552 | C=2; A=3; E=3 | C=0; A=0; E=0 |  |  |  |
|  |  |  |  |  |  |
|  |  |  |  |  |  |
| MGGC 8846/1UCC123a, MGGC 8846/1UCC123b, MPPPL 35, MPPPL 39 | C=3; A=3; E=n.a. | C=1; A=2; E=n.a |  |  |  |
|  |  |  |  |  |  |
|  |  |  |  |  |  |
| MM 25.5.1078 | C=0; A=n.a; E=n.a. |  |  |  |  |
|  |  |  |  |  |  |
|  |  |  |  |  |  |
| MGP-PD 27566 |  |  | C=2; A=1; E=1 |  | C=1; A=0; E=1 |
|  |  |  |  |  |  |
|  |  |  |  |  |  |
| MGP-PD 32438 | C=1; A=0; E=4 | C=2; A=2; E=4 |  |  |  |
|  |  |  |  |  |  |
|  |  |  |  |  |  |
| MCLSC T2 |  | C=2; A=2; E=4 | C=2; A=4; E=4 | C=0; A=0; E=4 | C=2; A=0; E=4 |
|  |  |  |  |  |  |

S2 Table: scoring for taphonomic values on the dataset. Scoring range from 0 to 4 (0% = 0; 1-25% = 1; 25-50% = 2; 50-75% = 3; 75-100% = 4); C = completeness (scoring for percentage of completeness for each anatomical unit); A = articulation (scoring for percentage of articulation for each anatomical unit); E = erosion (scoring for percentage of eroded compact bone for each anatomical unit).
